# Exploring*Ty* resistance genes and genetic diversity in improved tomato lines selected from commercial hybrids

**DOI:** 10.1186/s12870-025-07344-6

**Published:** 2025-09-23

**Authors:** Ahmed M.A. Mahmoud, Ahmed A. Hassan, Khaled E.A. Abdel-Ati, Neama H. Osman, Hassan A.A. Mohamed

**Affiliations:** 1 Vegetable Crops Department, Faculty of Agriculture,, Cairo University, Giza, 12613 Egypt; 2 Genetics Department, Faculty of Agriculture, Cairo University, Giza, 12613 Egypt

**Keywords:** Begomovirus, Fruit quality, Genotypic correlation, Multivariate analyses, Solanum lycopersicum

## Abstract

**Background:**

Tomato yellow leaf curl disease (TYLCD), caused by the *Tomato Yellow Leaf Curl Virus* (TYLCV), threatens global tomato production, making resistance breeding crucial. This study evaluated 17 tomato lines (TLs; F_6_ and F_7_) derived from F_1_ hybrids ‘65010’, ‘Nairouz’, ‘SVTD8320’, and ‘Tyrmes’ for 27 traits related to TYLCD resistance, vegetative growth, yield, and fruit quality under natural whitefly-mediated inoculation during the 2022 and 2023 fall seasons. Genetic diversity was assessed using phenotypic (PCV) and genotypic (GCV) coefficients of variation, heritability (*h*^*2*^_*b*_), and genotypic correlation coefficient (*r*_*g*_) of the estimated traits.

**Results:**

According to PCR-based specific markers, most TLs had multiple homozygous *Ty* resistance genes (> 2), except for TLs 1 and 3 (*Ty-4* gene only) and TL5 (no *Ty* genes). TLs exhibited high TYLCD tolerance, characterized by low symptom severity and viral replication, as observed in symptomless plants using TEM scanning. Most traits exhibited significant phenotypic variation across seasons, with 21 out of 27 traits showing high or moderate values of PCV (11.94–65.44%) and GCV (2.45–64.72%), and high *h*^*2*^_*b*_ (75.0-99.6%). This indicates that TLs have high genetic diversity and are suitable for phenotypic selection to improve TYLCD tolerance and productivity.

Significant estimates of *r*_*g*_ revealed that selecting for low TYLCD severity and high total yield, average fruit weight, fruit firmness, and fruit TSS content would lead to a reduction in fruit vitamin C content and the number of fruit locules. Conversely, this selection would increase early and marketable yield, number of plant fruits, fruit equatorial diameter, and fruit β-carotene content.

Principal component analysis reduced the 27 traits to five PCs, representing 85.07% of the total variance. Hierarchical cluster analysis classified TLs into four clusters, with Clusters 1 (TLs 1 and 5-12) and 3 (TL4 only) performing better for traits.

**Conclusion:**

Segregating commercial hybrid generations can be used to develop lines with multiple *Ty* resistance genes and enhanced traits. Crossing lines among these clusters could improve TYLCD tolerance and yield in future F_1_ hybrids. Some lines harbor TYLCD resistance genes, like TL5, which exhibited high TYLCD tolerance without detecting *Ty* genes. Further research is needed to identify the specific genes and mechanisms for resistance in these lines.

**Supplementary Information:**

The online version contains supplementary material available at 10.1186/s12870-025-07344-6.

## Background

Tomato yellow leaf curl disease (TYLCD), caused by the *Tomato Yellow Leaf Curl Virus* (TYLCV), is a major threat to global tomato production. TYLCV, a *Begomovirus* genus in the *Geminiviridae* family, is transmitted exclusively by the whitefly (*Bemisia tabaci* Genn.; Homoptera, Aleyrodidae). TYLCD symptoms include leaf yellowing and curling, stunted growth, flower absence, and fruit abortion, with early infections causing yield losses of up to 80–100% [1, 2]. TYLCD also diminishes fruit quality, resulting in smaller, deformed, and poorly colored fruits, thus reducing market value [1, 2]. The global spread of TYLCD has been driven by the invasive expansion of whiteflies, now reported in over 175 countries, with major outbreaks in around 70 [3, 4]. Climate factors, particularly rising temperatures and humidity, further exacerbate whitefly populations and virus transmission [5].

Controlling TYLCD primarily involves managing whiteflies, which is costly, labor-intensive, and increasingly ineffective due to insecticide resistance, high input costs, and environmental concerns [6–8]. Breeding TYLCD-resistant cultivars has become a more sustainable, practical, and eco-friendly alternative to mitigate yield losses and reduce virus spread [9, 10].

Resistance breeding efforts involve developing inoculation protocols, identifying and transferring resistance genes, and evaluating new lines. Natural whitefly-mediated inoculation is commonly used, as whiteflies are the only known TYLCV vectors [10–12]. Whiteflies can transmit TYLCV after 24 h of acquisition, with transmission efficiency reaching 100% when 5–15 whiteflies are present [13]. Genotypes are evaluated under natural infestation during peak whitefly activity seasons without insecticide application [9, 10, 12]. Resistance is assessed based on symptom severity, typically 30 days after transplanting (DAT), allowing susceptible plants to exhibit fully symptoms [2]. Symptomless plants or those with low viral titer (confirmed by PCR) are considered resistant, while those with mild symptoms are considered tolerant [10, 14–16].

TYLCD resistance was initially absent in cultivated tomato, but resistance has since been identified in wild relatives, as *S. chilense*, *S. habrochaites*, *S. peruvianum*, and *S. pennellii* [11, 16]. To date, six TYLCV resistance genes (*Ty-1* to *Ty-6*) have been mapped [16, 17]. *Ty-*1 and *Ty-3* dominant genes from *S. chilense* accessions LA1969 and LA2779 are mapped on chromosome 6 and associated with viral gene transcriptional silencing [18, 19]. *Ty-*2, another dominant gene from a tomato line H24 derived from *S. habrochaites* f. *glabratum* B6013, maps on chromosome 11 [20, 21], but is ineffective against some TYLCV strains [22, 23]. *Ty-4*, a dominant gene from *S. chilense* LA1932, is mapped on the long arm of chromosome 3 and confers lower resistance [24]. The recessive gene *ty-5*, from a TY172 tomato line originated from *S. peruvianum*, is mapped on chromosome 4 [25] and is linked to protein translation [26]. *Ty-6*, partially dominant from *S. chilense* LA1938 or LA2779, is mapped on the long arm of chromosome 10 [27]. Additionally, several quantitative trait loci (QTLs) have been identified. A QTL on chromosome 6 was identified in tomato ‘Rty Azur’ derived from *S. piminellifolium* hirsute INRA [28]. Line TY172 derived from *S. peruvianum* had four semi-dominant minor QTLs on chromosomes 1, 7, 9, and 11, respectively [29]. Line FLA 456, derived from *S. chilense* LA2779 and tomato ‘Tyking F_1_’, had four recessive QTLs (*qTy4.1*, *qTy6.1*, *qTy10.1*, and *qTy11.1*) on chromosomes 4, 6, 10, and 11 [30].

Initial tomato breeding efforts focused on introgressing single *Ty* genes, particularly *Ty-1*/*Ty-3* and *Ty-2*, from wild species into tomato cultivars, providing partial TYLCD resistance [31]. However, TYLCV-resistance-breaking strains have emerged [22, 32, 33], the *Ty-2* gene is ineffective against many global TYLCV strains [23], and *Ty-4* offers limited resistance [30]. Advances in molecular biotechnology have enabled the precise mapping of resistance loci and the development of gene-specific molecular markers, facilitating the pyramiding of multiple *Ty* genes in tomato cultivars. This approach enhances resistance across diverse viral strains and extends the longevity of resistant cultivars [16, 17]. Consequently, several commercial F_1_ hybrids with multiple resistance genes, such as ‘Dania’, ‘Brivio’, ‘SVTD8320’, and ‘Tyrmes’ (harboring *Ty-*1/*Ty-*3, *Ty-*2/*Ty-*2, *Ty-4*/*Ty-4*, and *Ty-5*/*ty-5*), have been released by seed companies [12, 17, 31].

TYLCD-resistant F_1_ hybrids with high yield and fruit quality have become valuable sources of resistance in breeding programs [9, 12]. Segregating generations from these hybrids has enabled the successful selection of resistant/tolerant lines [5, 10, 34–36]. Moustafa et al. [34] developed F_7_ TYLCD-resistant tomato lines with good fruit quality from commercial hybrids ‘Fiona’ and ‘Tyking’. El-Morsy et al. [35] selected six TYLCD-resistant F_5_ lines from hybrids ‘DANYA’, ‘6130’, ‘3017’, ‘783-N’, ‘TYG’, and ‘KIS-N’. Mahmoud and Osman [10] identified 12 F_9_ lines from commercial hybrids ‘TH99802’ and ‘TH99806’, which exhibited TYLCD tolerance with vigorous growth and high yield. Nguyen et al. [36] selected ten elite F_8_ lines via bulk selection, exhibiting distinctive superior agronomical traits, including early maturity, high fruit set and yield, jointless pedicels, partial parthenocarpy, and inclusion of *Ty-1*/*Ty-3* loci. However, Koeda et al. [5] stated that homozygous resistance genes, particularly *Ty-1*/*Ty-3*/*Ty-3a*, can increase TYLCD resistance but may negatively affect yield and fruit quality due to linkage drag. Therefore, ongoing breeding efforts are essential to maintain resistance durability while meeting commercial standards for flavor, appearance, and productivity.

Plant breeders commonly use morphological traits to assess genetic variability, as they are easy to score, quick to analyze, and cost-effective [10]. For selecting TYLCD-resistant/tolerant plants, traits related to vegetative growth, productivity, and fruit quality, often affected by TYLCV infections, should be prioritized. Genetic diversity is evaluated through several statistical methods, such as phenotypic and genotypic coefficients of variation (PCV and GCV) and multivariate analyses, particularly principal component analysis (PCA) and cluster analysis (CA). Furthermore, estimates of heritability (*h*^*2*^_*b*_) and genetic correlations are essential, as they provide insight into the potential for effective selection. To develop tomato lines with multiple *Ty* genes, Mohamed (2021) [37] selected several TYLCD-symptomless F_3_ plants with desirable agronomic traits from commercial F_1_ hybrids ‘SVTD8320’, ‘Tyrmes’, ‘Nirouz’, and ‘65010’, which carried multiple *Ty* genes [12]. Building on this effort, the present study evaluated 17 improved tomato lines (TLs; F_6_ and F_7_ generations) during the 2022 and 2023 fall seasons under natural whitefly-mediated infection. The lines were evaluated for TYLCD resistance, vegetative growth, productivity, and fruit quality traits. Genetic diversity was analyzed using *PCV*, *GCV*, *h*^*2*^_*b*_, and multivariate analyses. Furthermore, *Ty* genes were identified using gene-specific PCR markers.

## Materials and methods

### Tomato lines

A bulk selection breeding program was initiated in 2017 to develop TYLCD-resistant tomato lines from resistant commercial F_1_ tomato hybrids: ‘Nairouz’, ‘65010’, ‘SV8320’, and ‘Tyrmes’ [37]. Selections were performed during the fall seasons under natural viruliferous whitefly field infestation at the Agricultural Experiment Station (AES), Giza, Egypt (30°01′07′′N; 31°12′28′′E). Seventeen out of 40 symptomless F_5_ plants were selected based on average fruit weight > 90 g, total plant yield > 3000 g, fruit TSS content > 3.6 °Brix, and fruit firmness > 4.5kg cm^−2^ (Table S1). These selections were advanced to the F_6_ and F_7_ generations (referred to as tomato lines; TLs) for evaluation of TYLCD resistance, vegetative growth, productivity, and genetic diversity.

### Experimental procedures

#### Planting

Seventeen TLs were evaluated under natural viruliferous whitefly field infestation at the AES during the 2022 and 2023 fall seasons. Seeds were sown on the 1 st of July in both seasons in seedling trays filled with a mixture of coconut peat and vermiculite (volume 1:1). Five-week-old seedlings were field-transplanted in a randomized complete block design (RCBD) with three replicates. Transplants were spaced 50 cm apart in two rows (1 × 3 m) per line for each experimental unit (EU). Standard agricultural practices were followed without applying insecticides.

#### Whitefly-mediated natural inoculation

**Fig. 1:**
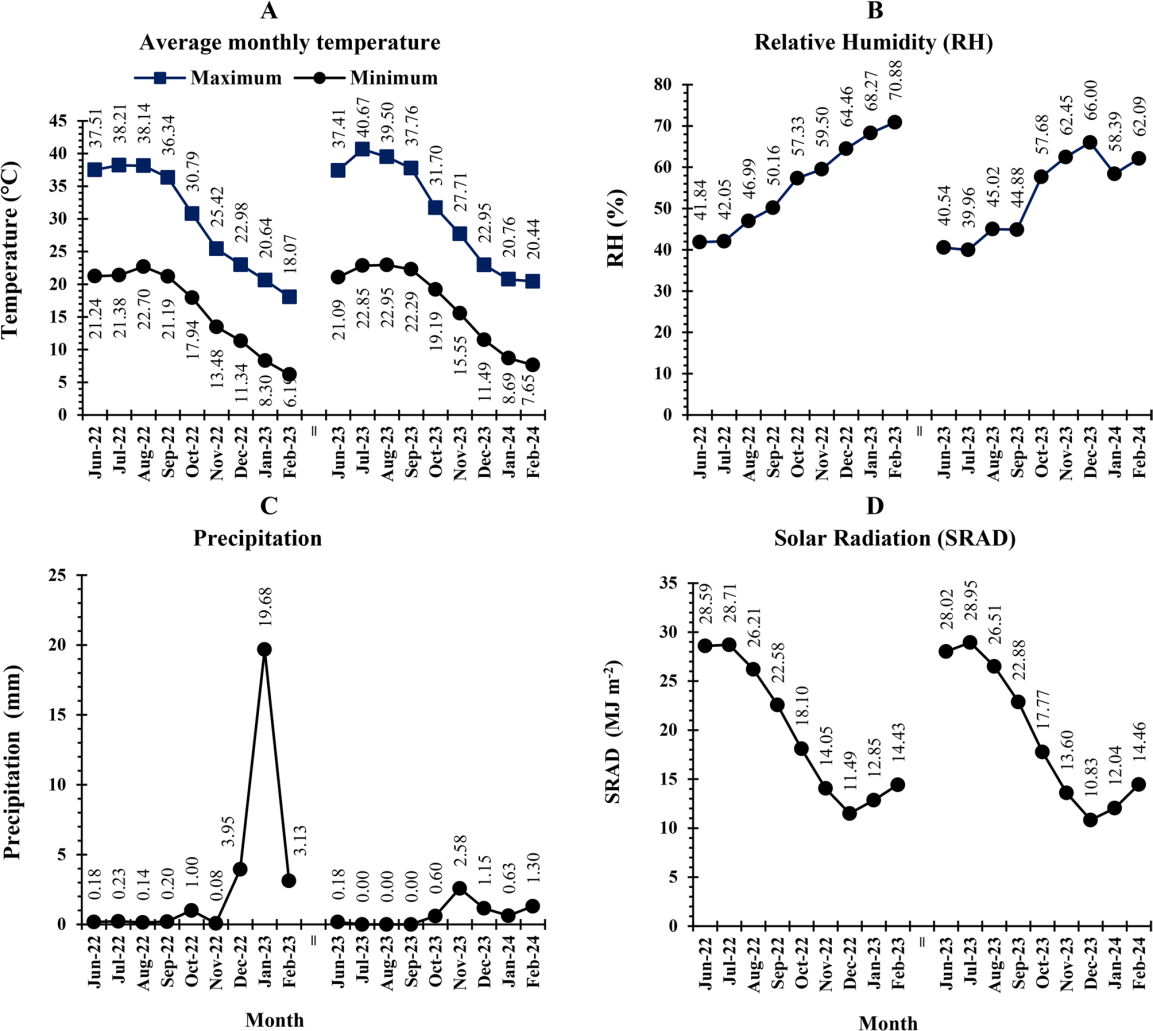
Meteorological data from June to February in the 2022 and 2023 seasons. **A:** minimum and maximum temperatures, **B:** relative humidity; **C:** precipitation, and **D**: Solar radiation. Source: Central Laboratory for Agricultural Climate (CLAC), Agricultural Research Center, Ministry of Agriculture and Land Reclamation, Egypt

Viruliferous whiteflies flourish in Egypt from April to November, with a peak in August to October [38]. The use of natural whitefly infestations for viral inoculation in this study was based on their prevalence in nurseries and fields, influenced by the climate during both seasons, as shown in Fig. 1. Insecticides were not used to encourage the spread of whiteflies in these areas [10].

### Detection of TYLCV resistance genes

Tomato lines with known *Ty* resistance genes used as positive controls: LA3473 (*Ty-1*), CLN2498E (*Ty-2*), LA4440 (*Ty-3* and *Ty-4*), and FLA456 (*ty-5 & Ty-6*), while the ‘Castlerock’ served as a susceptible negative control. DNA was extracted from 3-7-day-old seedlings using the Cetyltrimethylammonium bromide method [39] and adjusted to 10 ng µl^−1^. PCR assays were performed using Eppendorf^®^ Mastercycler Gradient 5 (Hamburg, Germany). *Ty* genes were detected in TLs using CAPS and SCAR gene-based markers presented in Table 1. Each PCR reaction (25 µl) included 2.5 µl 2.5 mM dNTPs, 5 µl 5× PCR buffer, 2.5 µl 2.5 mM MgCl_2_, 0.1 µl (0.5 units) Taq DNA polymerase, 2.5 µl each forward and reverse primer at 10 µM, 2–5 µl of DNA extract, and ddH_2_O to final volume. PCR cycles involved 94 °C for 2–4 min, 35 cycles of 94 °C for 30–40 s, 50–55 °C (depending upon the specific melting temperature of the primers) for 40–60 min, and 72 °C for 1–1.5 min. A final elongation step was at 72 °C for 5–10 min; then the reaction was held at 4 °C with only CAPS markers. The PCR-amplified fragments were separated by gel electrophoresis with 2% agarose in 0.5× tris/borate/EDTA buffer (89 mM Tris-HCl,89 mM boric acid, 2.5 mM EDTA, pH 8.3), stained with ethidium bromide, and visualized with UV light. Approximately 10 µl of the PCR reaction was used in a 25 µl reaction mixture with *Taq-1* for all CAPS markers, except for C2_At4g17300, which uses *AfII* restriction enzymes (Promega Corp.) at 65 °C for 2–4 h. Fragments separated on 2% agarose gels in 0.5× TBE buffer, stained with ethidium bromide, and visualized with UV light.

**Table 1:** Sequences of the used primers

Marker	Marker type	Targeted gene	Chromosome number	Single nucleotide sequence (5’−3’)	Annealing temperature (°C)	Restriction enzyme	Molecular size of bands (bp)	Molecular size after digesting with enzyme (bp)	Reference
TY-1/3_K	**SCAR**	***Ty-1*** **/** ***Ty-3***	**6**	F: ACAGGAAAAATGGGTGATCC R: CCTGCTCCTTGCAGATTCTA	55	-	RR: 114 SS: 102	-	**Chen et al.** [40]
Rex-1	**CAPS**	***Ty-1***	**6**	F: TCGGAGCCTTGGTCTGAATT R: ATGCCAGAGATGATTCGTGA	55	*Taq-1*	750	RR: 570&180 SS: 750	**Williamson et al.** [41]
TY-1	**CAPS**	***Ty-1***	**6**	F: TAATCCGTCGTTACCTCTCCTT R: CGGATGACTTCAATAGCAATGA	55	*Taq-1*	398	RR: 300&95 SS: 398	**Chen et al.** [42]
P6-25	**SCAR**	***Ty-3*** ***Ty-3a*** ***Ty-3b***	**6**	F: GGTAGTGGAAATGATGCTGCTC R: GCTCTGCCTATTGTCCCATATATAACC	53	-	RR: 623 bp for *Ty-3a*, 453 bp for *Ty-3*, and 660 bp for *Ty-3b.* SS: 320 bp	-	**Nevame et al.** [43]
FER-G8	**CAPS**	***Ty-3***	**6**	F: CATCCCGTGCATCCAAAGTGAC R: CTAAGGGTGTACCCCAAGGGAAC	55	*Taq-1*	500	RR: 50, 200, 250, 300 & 500	**Ji et al.** [20]
TG0302	**SCAR**	***Ty-2***	**11**	F: TGGCTCATCCTGAAGCTGATAGCGC R: AGTGTACATCCTTGC CATTGACT	55	-	RR: 900 SS: 800	-	**Garcia et al.** [44]
C2_At4g17300	**CAPS**	***Ty-4***	**3**	F: ATTTAACCGTGTCTGGGCAACTCAATGG R: GCTCACTTTGCAAATCACATCCCCATTTCACC	55	*Afll*	325	RR: 325 SS: 200	**Ji et al.** [24]
SINAC1	**CAPS**	***ty-5***	**4**	F: TGCCTGGTTTCTGCTGTCA R: TAAAGCTGAAGAAGGACTTACCCT	55	*Taq-1*	420	RR: 300/425 SS: 350	**Anbinder et al.** [29]
UF_10.61192	**CAPS**	***Ty-6***	**10**	F: CATAAAGTTCCGGCGAGTGT R: TCCATTCCAAACCAAGTGAAG		*BssHII*	632	RR: 632 SS: 600	**Gill et al.** [27] **andSim et al.** [45]

### Evaluation of TYLCD resistance/tolerance

TYLCD resistance/tolerance was evaluated in TLs at 45 and 90 DAT across both seasons by scoring symptom severity on a 1–5 scale, following Mahmoud et al. [12] and shown in Figure S1. TYLCD symptoms severity (TYLCDS) for each line was calculated based on the individual plant ratings using the following equation: TYLCDS = $\:\frac{\sum\:(\text{N}\text{u}\text{m}\text{b}\text{e}\text{r}\:\text{o}\text{f}\:\text{p}\text{l}\text{a}\text{n}\text{t}\text{s}\:\text{o}\text{f}\:\text{e}\text{a}\text{c}\text{h}\:\text{g}\text{r}\text{a}\text{d}\text{e}\:\times\:\text{d}\text{i}\text{s}\text{e}\text{a}\text{s}\text{e}\:\text{g}\text{r}\text{a}\text{d}\text{e})}{\text{T}\text{o}\text{t}\text{a}\text{l}\:\text{n}\text{u}\text{m}\text{b}\text{e}\text{r}\:\text{o}\text{f}\:\text{p}\text{l}\text{a}\text{n}\text{t}\text{s}}$.

### Detection of TYLCV molecules by transmission electron microscopy

Leaf samples from symptomless plants of TL6 and TL7, along with symptomatic plants of ‘Castlerock’, TL10, TL15, and TL16 at 90 DAT, were examined using a transmission electron microscope (TEM) to characterize viral molecules in cells. The fully extended plant leaves were collected from the experimental field, packed in plastic bags with moist tissue paper, and prepared for TEM analysis. Plant tissue samples from petioles or veins were processed using standard procedures described by Cherif and Russo [46]. Samples were fixed for 2 h in 4% glutaraldehyde in 0.05 M cacodylate buffer, pH 7.0, under a slight vacuum at room temperature, then rinsed in the buffer. Samples were post-fixed for 2 h at 4 °C in 1% (w/v) osmium tetroxide in cacodylate buffer, stained overnight at 4 °C in 0.5% uranyl acetate, dehydrated in graded ethanol dilutions, and embedded in Spurr’s medium. Thin sections were double-stained with uranyl acetate and lead citrate and examined under a JEM-1400 transmission electron microscope (JEOL, Tokyo, Japan) at 80 kV with images captured by a side-mounted CCD digital camera (EMT Optronics, 1632 × 1632-pixel format) at magnifications of 80,000- 120,000X.

### Evaluation of plant vegetative and productivity traits

A successful horticultural cultivar must combine consumer-preferred traits with economic viability for producers. TYLCD infection adversely affects plant growth, productivity, and fruit quality by damaging the foliage [1]. Therefore, in addition to disease resistance, traits related to vegetative growth, yield components, and fruit quality were evaluated in the TLs.

#### Vegetative traits

Vegetative traits related to plant canopy area, *namely*, plant length (PL), number of plant main branches (NPMB), and the area of the fifth leaf from the apex (LA), were measured 90 DAT on five randomly selected plants from each EU, excluding plants from row edges. Leaf photosynthesis pigments content (chlorophyll a: chlor-a, chlorophyll b: chlor-b, total chlorophyll: t-chlor, and total carotenoids: t-carot) was extracted by dimethylformamide and estimated according to Moran [47]. According to Carmassi et al. [48], LA was estimated using the length (L) and width (W) of the leaf, and the equation. LA = $\:\sqrt{L\times\:W}$. LA is leaf area, L is leaf length, and W is leaf width.

#### Fruit yield and quality traits

The plant’s early (EY), total (TY), and marketable (MY) yields were estimated. The weight of all collected fruits from all harvests was used to estimate TY, while the weight of the first two harvests was used to estimate EY. To estimate MY, fruits with cracks, blossom-end rot, external watery transparent tissue, and fruits weighing less than 30 g were excluded from TY. The average fruit weight (AFW) was estimated as the mean weight of all the collected normal fruits of the plant. The number of fruits/plant (NPF) was counted from all the collected plant’s marketable fruits.

Fruit’s quality traits were evaluated by harvesting 20 fully red-ripe fruits from each EU during the peak harvesting period and washing them with distilled water. The fruit’s physical qualities were measured by their polar (FPD) and equatorial (FED) diameters, fruit shape index (FSI), number of fruit locules (NFL), flesh thickness (FT), and fruit firmness (FF). The FSI was calculated as the ratio of the polar to equatorial diameter. According to Yeager [49], the oval fruit shape has a ratio of 1.2 or more, the round shape ratio is 0.95–1.19, and the oblate shape ratio is less than 0.95. FF was measured using a Mark-10 (Series 4) pressure tester (Force Gauge Model M4-200) equipped with a 1 mm flat probe.

Chemical quality included the content of total soluble solids (TSS), titratable acidity (TA), vitamin C (VC), lycopene (Lyc), and β-carotene (β-Car). TSS was measured using a digital refractometer (PR101, Palette Co. Ltd., Tokyo, Japan). TA was ascertained using a 0.1 N NaOH solution and phenolphthalein as an indicator [50]. VC content was estimated according to Tareen et al. [51], by blending 5 g of fruit pulp with 5 ml of 1.0% (w/v) hydrochloric acid, and centrifuging at 10,000x for 10 min, measuring the absorbance of the supernatant at 243 nm using a Jenway 6305 UV/visible spectrophotometer, and calibrating with standard VC solutions. Lycopene and ß-Carotene content of pericarp samples were determined according to Sánchez-González et al. [52].

As indicators of tomato taste and flavor, the taste index (TI) and maturity (M) were also estimated to assess consumer acceptance and distinguish between lines, as mentioned in previous papers [53, 54]. Equations provided by Navez et al. [55] were used to calculate TI and M as follows TI = $\:\frac{^\circ\:\text{B}\text{r}\text{i}\text{x}\:\text{d}\text{e}\text{g}\text{r}\text{e}\text{e}}{\left(20\times\:\text{T}\text{A}\right)+\text{T}\text{A}}$ and M = $\:\frac{^\circ\:\text{B}\text{r}\text{i}\text{x}\:\text{d}\text{e}\text{g}\text{r}\text{e}\text{e}}{\text{T}\text{A}}$, where °Brix is the TSS value and TA is the titratable acidity value. According to Hernández Suárez et al. [52, 53] and Navez [54], tomato fruits with TI values > 0.7 and maturity > 10 were considered tasty.

### Biometrical analyses

#### Analyses of variance and mean comparisons

Phenotypic data were tested for normality using the Shapiro—Wilk test. Data for TYLCDS, EY, TY, AFW, FSI, TA, TI, and M were transformed using arcsine square root [56]. ANOVA was performed using a RCBD over both seasons. Significant differences between the means were determined using Duncan’s multiple range test at a 5% probability level. ANOVA and mean comparisons were conducted using MSTATc v.2.1 (Michigan State University, Michigan, USA).

#### Genetic parameters

A combined ANOVA over both seasons was used to estimate variance components related to differences among TLs, including genotypic (*δ*^2^_g_), phenotypic (*δ*^2^_ph_), genotype × year (*δ*^2^_gy_), and pooled error (*δ*^2^_e_) variance components, following the equations stated by Singh and Chaudhary [57], as presented in Table S2. The GCV and PCV were estimated according to Burton [58] as follows: GCV= $\:\frac{\sqrt{{\delta\:}_{g}^{2}}}{\stackrel{-}{x}}\times\:100$ and PCV= $\:\frac{\sqrt{{\delta\:}_{ph}^{2}}}{\stackrel{-}{x}}\times\:100$, where $\:\stackrel{-}{x}$ is the grand mean of the trait. The GCV and PCV are classified as low (< 10%), moderate (10–20%), and high (> 20%) as suggested by Johnson et al. [59]. Broad sense heritability (*h*^2^_b_) for a trait was estimated according to Johnson et al. [59], as follows: ***h***^2^_b_ = $\:\frac{{\varvec{\delta\:}}_{\varvec{g}}^{2}}{{\varvec{\delta\:}}_{\varvec{p}\varvec{h}}^{2}}\times\:100$. The *h*^2^_b_ is classified as low (< 30%), moderate (30–60%), and high (> 60%). Covariance components were substituted to compute *r*_*g*_ and *r*_*ph*_ according to Johnson et al. [59] and Singh and Chaudhary [56]. *r*_*g*_= $\:\frac{{COV}_{{g}_{\left(x1x2\right)}}}{\sqrt{{\delta\:}_{{g}_{\left(x1\right)}}^{2}{\delta\:}_{{g}_{\left(x2\right)}}^{2}}}$, where $\:{COV}_{{g}_{\left(x1x2\right)}}$ is the genotypic covariance between a given pair of traits (x1 and x2) and $\:{\delta\:}_{{g}_{\left(x1\right)}}^{2}$ and $\:{\delta\:}_{{g}_{\left(x2\right)}}^{2}$ are the genotypic variances of x1 and x2, respectively. *r*_*ph*_= $\:\frac{{COV}_{{ph}_{\left(x1x2\right)}}}{\sqrt{{\delta\:}_{{ph}_{\left(x1\right)}}^{2}{\delta\:}_{{ph}_{\left(x2\right)}}^{2}}}$, where $\:{COV}_{{ph}_{\left(x1x2\right)}}$ is the phenotypic covariance between two traits (x_1_ and x_2_) and *δ*^*2*^_*ph*(*x1*)_ and *δ*^*2*^_*ph*(*x2*)_ are phenotypic variances of x1 and x2, respectively. The significance of *r*_*g*_ and *r*_*ph*_ was tested based on the standard error of the correlation coefficient between two traits according to Yassin [60].

#### Multivariate analyses

Scale disparities were minimized by standardizing the pooled data for each trait using Z-scores before performing multivariate analyses. PCA was performed with Varimax rotation [61], and parallel analysis was used along with the latent root criterion (eigenvalue > 1) to identify statistically significant components [62]. A biplot was created between the first two PCs, which sufficiently described a sizable portion of the overall variation, to help determine the links between PCs and traits and lines, lines and traits, and inter-trait [63]. The correlation between traits was estimated by calculating the cosine of the angle between the vectors, which can indicate independence (= 90◦), positive (< 90◦), or negative correlation (> 90◦) [63]. Hierarchical cluster analysis (HCA) was employed using square Euclidean distance and Ward’s joining technique to create a dendrogram. All multivariate statistical analyses were performed using IBM SPSS version 26.0.0 (SPSS Inc., Chicago, IL) and XLSTAT software version 2019 (Addinsoft, Paris, France).

## Results

### The presence of TYLCD resistance genes in tomato lines

**Fig. 2:**
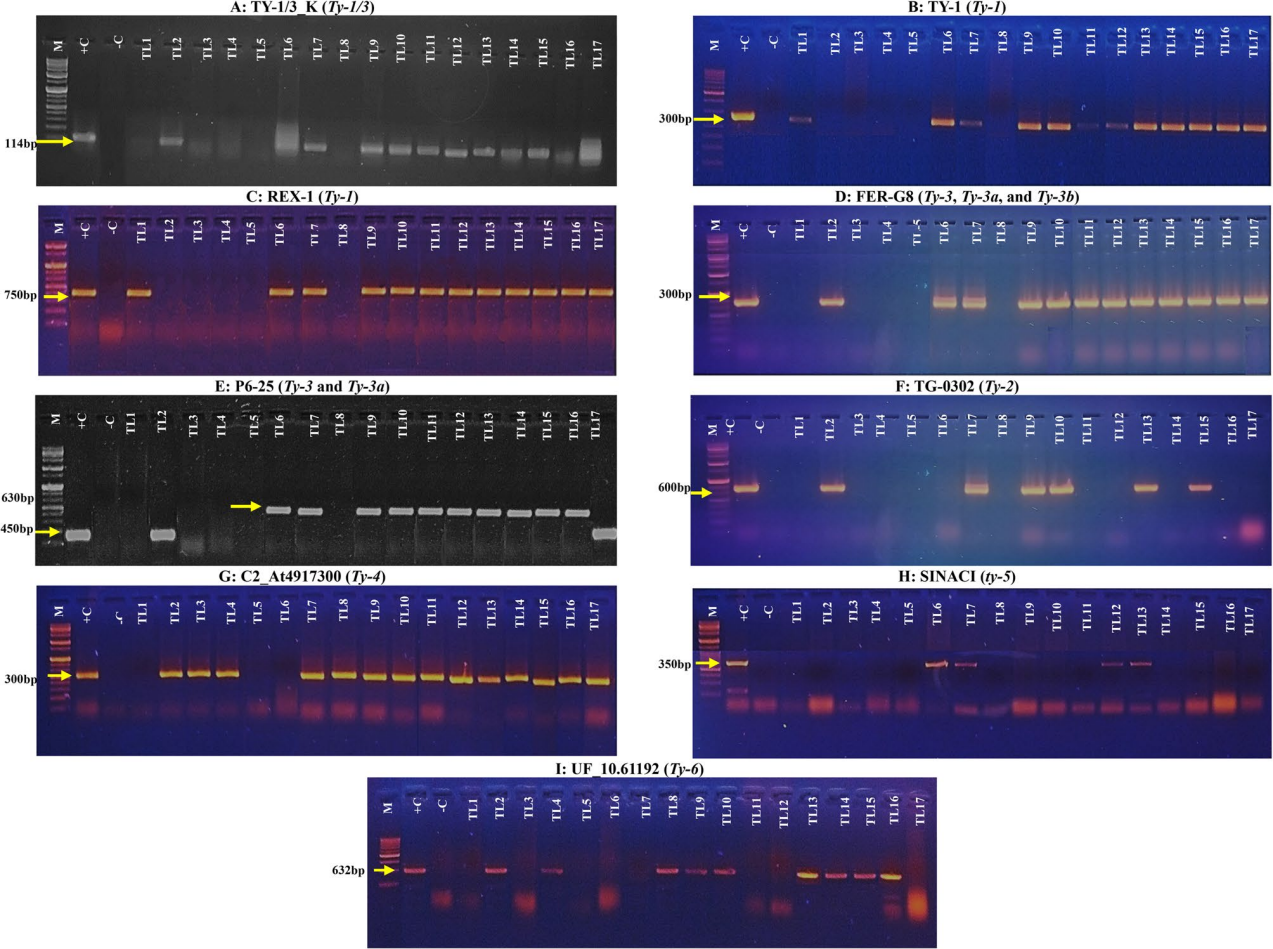
The presence of *Ty* genes in tomato lines based on PCR-based specific markers. Markers were TY-1/3_K for *Ty-1*/*Ty-3* gene (**A**), TY-1 and Rex-1 for *Ty-1* allele (**B**&**C**), Fer-G8 and P6-25 for *Ty-3* allele (**D**&**E**)*,* TG-0302 for *Ty-2* gene (**F**), C2_At4g17300 for *Ty-4* gene (**G**), SINACI for *ty-5* gene (**H**), and UF10.61192 for *Ty-6*gene (**I**). Lanes M: 1kbp DNA marker, +C: LA3473 for *Ty-1*, CLN2498 for *Ty-2*, LA4440 for *Ty-3* and *Ty-4*, and FL456 for *ty-5* and *Ty-6*,-C: susceptible ‘Castlerock’, TL1&TL2: F_7_ lines of ‘Nairouz F_1_’, TL3 - TL6: F_7_ lines of ‘65010 F_1_’, TL7 - TL16: F_7_ lines of ‘SVTD8320 F_1_’, and TL17: F_7_ of ‘Tyrmes F_1_’

The primer pair TY-1/3_K amplified fragments of a 102 bp amplicon for ‘Castlerock’, TL1, TL3, TL4, TL5, and TL8, and a 114 bp amplicon for the remaining TLs (Fig. 2A). After *Taq-1* digestion, TY-1 primer amplified fragments of a 398 bp amplicon for ‘Castlerock’, TL2, TL3, TL4, TL5, and TL8, and a 300 bp amplicon for the other TLs (Fig. 1B). REX-1 primer, after *Taq-1* digestion, generated fragments of a 570 bp amplicon for ‘Castlerock’, TL2, TL3, TL4, TL5, and TL8, and a 750 bp amplicon for the other TLs (Fig. 1C). FER-G8 primer, after *Taq-1* digestion, amplified fragments of a 200 bp amplicon for ‘Castlerock’, TL1, TL3, TL4, TL5, and TL8, a 300 bp amplicon for TL6-TL7 and TL9-TL16 (Fig. 1D). P6–25 primer amplified a fragment of a 350 bp amplicon for ‘Castlerock’, TL1, TL3, TL4, TL5, and TL8, a 450 bp amplicon for TL2 and TL17, and a 630 bp amplicon for TL6-TL7 and TL9-TL16 (Fig. 1E). TG0302-SCAR primer amplified a 450 bp amplicon for ‘Castlerock’, TL1, TL3-TL6, TL8, TL11-TL12, TL14, and TL16-TL17, and a 600 bp amplicon for TL2, TL7, TL9-TL10, TL13, and TL15 (Fig. 1F). C2_At4g17300 primer, after *Afll* digestion, amplified a 200 bp fragment for all TLs, except for TL4-TL5 and ‘Castlerock’, which showed a 200 bp amplicon. (Fig. 1G). SINACI primer amplified a 350 bp amplicon for all TLs, except TL6-TL7, TL12-TL13, and ‘Castlerock’, which amplified a 425 bp amplicon (Fig. 1H). UF_10.61192 primer, after *BssHII* digestion, amplified fragments of a 632 bp for TL2, TL4, TL8-TL10, and TL13-TL16, and a 600 bp amplicon for the other TLs and ‘Castlerock’ (Fig. 1I).

### Phenotypical characterization

#### ANOVA

Table 2 presents the combined ANOVA results for 27 traits across the 2022 and 2023 fall seasons for 17 tomato lines, along with ‘Castlerock’. Significant differences across years (*MS*_*y*_) were only found with 12 of the 27 evaluated traits, with *P* < 0.001 for EY, TY, MY, FT, Lyc; *P* < 0.01 for NPF, NFL, and TA; and *P* < 0.05 for PL, PD, TSS, and VC.

**Table 2:** The combined ANOVA and genetic parameters for 27 traits of tomato lines and ‘Castlerock’ over the 2022 and 2023 fall seasons

**Trait**^z^	**Source of Variance**							
	**Year (Y)df= 1**		**Replication (Y)df= 4**	**Genotypes (G)df= 17**		**Y × Gdf= 17**	**Pooled Errordf= 68**	
TYLCDS-45	0.564^ns^		0.06	2.091^***^		0.070^ns^	0.049	1.79
TYLCDS-90	1.181^ns^		0.198	3.419^***^		0.308^**^	0.113	2.1
PL	105.613^*^		3.843	9525.423^***^		41.135^***^	4.882	87.55
NPMB	0.113^ns^		0.151	10.904^***^		0.073^ns^	0.283	6.88
LA	30619.233^ns^		10730.59	23732.969^***^		2944.508^ns^	1905.141	279.31
Chlor-a	404.802^ns^		732.705	74.048^ns^		34.934^ns^	143.488	29.41
Chlor-b	305.175^ns^		66.566	21.071^ns^		13.579^ns^	26.312	12.42
T-Chlor	1412.829^ns^		1139.387	165.835^ns^		84.266^ns^	286.578	41.83
T-Carot	80.696^ns^		32.934	3.304^ns^		1.765^ns^	5.654	5.1
NPF	1085.535^**^		5.509	409.882^***^		23.948^***^	0.846	34.8
AFW	57.985^ns^		2.675	1423.126^***^		157.405^***^	7.166	94.48
EY	4704181.481^***^		1671.815	1384739.678^***^		48425.599^***^	12671.84	1197.3
TY	11507819.593^***^		61210.572	4471028.318^***^		192030.377^***^	5258.366	3360.41
MY	17698908.521^***^		119525.52	4079444.989^***^		198097.335^***^	19386.39	3086.69
PD	0.467^*^		0.043	0.256^***^		0.179^***^	0.013	4.62
ED	0.636^ns^		0.141	1.702^***^		0.228^***^	0.019	5.69
FSI	0.000077^ns^		0.0005	0.080^***^		0.007^***^	0.000324	0.82
FF	0.191^ns^		0.017	0.353^***^		0.288^***^	0.008	3.98
NFL	0.593^**^		0.001	4.671^***^		1.088^***^	0.001	4.2
FT	0.339^***^		0.0005	0.033^***^		0.022^***^	0.000294	0.55
TSS	1.235^*^		0.043	0.302^***^		0.046^ns^	0.029	5.07
VC	6.811^*^		0.095	69.215^***^		35.380^***^	0.062	14.34
TA	0.076^**^		0.003	0.055^***^		0.006^**^	0.002	0.5
Lyc	0.109^***^		0.001	0.068^***^		0.012^***^	0.004	0.43
ß-Carot	0.002^ns^		0.001	0.006^***^		0.002^***^	0.001	0.11
TI	0.012^ns^		0.002	0.011^***^		0.002^ns^	0.001	1.04
M	10.867^ns^		2.749	33.814^***^		4.122^*^	2.118	10.7

	Genetic parameters							
	*δ*^2^_g_	*δ*^2^_gy_	*δ*^2^_e_	*δ*^2^_ph_	*ECV* (%)	*PCV *(%)	*GCV *(%)	*h*^2^_b_(%)
TYLCDS-45	0.337	0.007	0.049	0.349	12.36	32.96	32.40	96.65
TYLCDS-90	0.519	0.065	0.113	0.570	16.04	36.01	34.35	90.99
PL	1580.715	12.08	4.882	1587.571	2.52	45.51	45.41	99.57
NPMB	1.805	-0.07	0.283	1.817	7.73	19.58	19.52	99.33
LA	3464.744	346.5	1905	3955.495	15.63	22.52	21.07	87.59
Chlor-a	6.519	-36.2	143.5	12.341	40.73	11.94	8.68	52.82
Chlor-b	1.249	-4.24	26.31	3.512	41.31	15.09	9.00	35.56
T-Chlor	13.595	-67.4	286.6	27.639	40.67	12.57	8.81	49.19
T-Carot	0.257	-1.3	5.654	0.551	46.69	14.56	9.93	46.58
NPF	64.322	7.701	0.846	68.314	2.64	23.75	23.05	94.16
AFW	210.954	50.08	7.166	237.188	2.83	16.3	15.37	88.94
EY	222719.013	11918	12672	230789.946	9.40	40.12	39.42	96.50
TY	713166.324	62257	5258	745171.386	2.16	25.69	25.13	95.71
MY	635084.773	63106	19386	669868.593	4.51	26.52	25.82	94.81
PD	0.013	0.055	0.013	0.043	2.47	4.47	2.45	30.08
ED	0.246	0.07	0.019	0.284	2.42	9.36	8.71	86.60
FSI	0.012	0.002	0	0.013	2.19	14.03	13.4	91.25
FF	0.011	0.093	0.008	0.059	2.25	6.09	2.61	18.41
NFL	0.597	0.362	0.001	0.779	0.75	20.99	18.38	76.71
FT	0.002	0.007	0	0.006	3.10	13.41	7.74	33.33
TSS	0.043	0.006	0.029	0.050	3.36	4.42	4.07	84.77
VC	5.639	11.77	0.062	11.536	1.74	23.68	16.56	48.88
TA	0.008	0.001	0.002	0.009	8.96	19.19	18.11	89.09
Lyc	0.009	0.003	0.004	0.011	14.67	24.7	22.42	82.35
ß-Carot	0.001	0	0.001	0.001	28.23	32.6	28.23	75.00
TI	0.002	0	0.001	0.002	3.06	4.14	3.74	81.82
M	4.949	0.668	2.118	5.636	13.6	22.18	20.79	87.81

All estimated traits showed highly significant differences (*P* < 0.001) among genotypes (*MS*_*g*_), except for foliar photosynthesis pigment content (Table 2). The mean squares due to G×Y interaction (*MS*_*gy*_) had significant effects on 19 out of 27 traits. No significant differences were seen in *MS*_*gy*_ of TYLCD-S-45, TYLCD-S-90, NPB, LA, Chlor-a, Chlor-b, T-Chlor, T-Car, TSS, and TI.

#### TYLCV resistance/tolerance

**Fig. 3:**
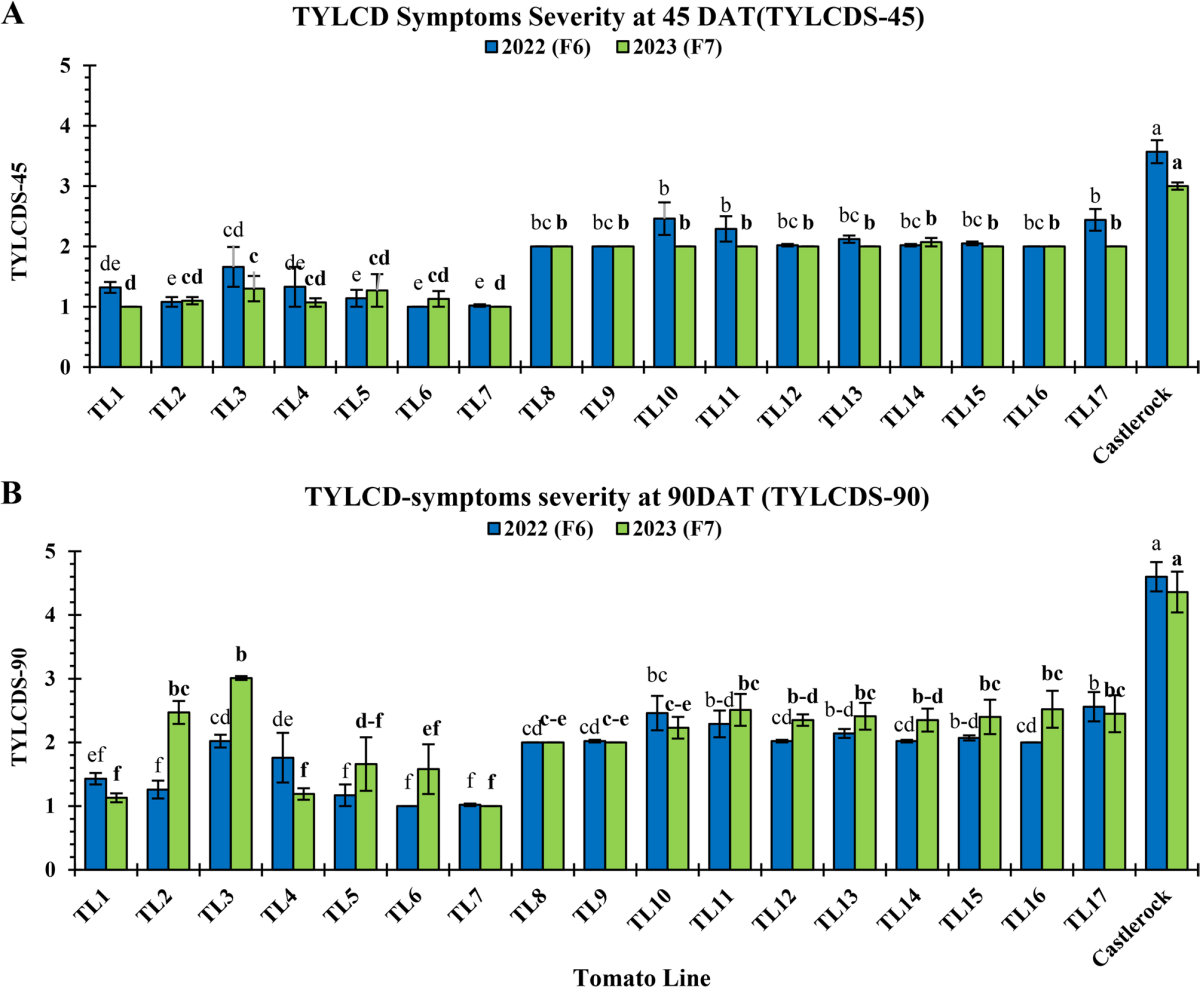
TYLCD symptoms severity in tomato lines and ‘Castlerock’ at 45 (**A**) and 90 (**B**) days after transplanting. Lines were F_6_& F_7_ generations from tomato commercial F_1_ hybrids ‘Nairouz’ for TL1&TL2, ‘65010’ for TL3&TL6,‘SVTD8320’ for TL7 - TL16, and ‘Tyrmes’ for TL17. TYLCD severity was estimated based on a 1-5 scale, where 1: symptomless, 2: slight, 3: moderate, 4: severe, and 5: very severe symptoms. TYLCDS data were transformed by the arcsin equation for statistical analysis. Columns with the same letter represent values that are not significantly different at the 5% probability level according to Duncan’s multiple range test. Vertical bars represent ± standard error of the means (replicates = 3)

TYLCD symptom severity scores (TYLCDS) for TLs at 45 (TYLCDS-45) and 90 DAT (TYLCDS-90) are presented in Fig. 3. TYLCDS-45 scores ranged from 1.00 to 3.57 in 2022 and 1.00 to 3.00 in 2023 (Fig. 3A), while TYLCDS-90 scores ranged from 1.00 to 4.60 in 2022 and 1.00 to 4.36 in 2023 (Fig. 3B). Across both generations, TLs generally exhibited mild to moderate symptoms, with TYLCDS-45 scores ranging from 1.00 to 2.46 in F_6_ and from 1.00 to 2.07 in F_7_ (Fig. 3A), and TYLCDS-90 scores ranging from 1.00 to 2.56 in F_6_ and 1.00 to 3.01 in F_7_ (Fig. 3B). TL1 and TL5-TL7 consistently showed significantly lower (*P* < 0.05) TYLCDS-45 scores (ranging 1.00–1.32 in F_6_ and 1.00–1.27 in F_7_; Fig. 3A) and TYLCDS-90 scores (ranging 1.00–1.43 in F_6_ and 1.00–1.66 in F_7_; Fig. 3B), with significant similarities (*P* < 0.05) among them and to TL2 and TL4 for TYLCDS-45 only in both generations (Fig. 3A). In both seasons, the susceptible ‘Castlerock’ exhibited significantly higher TYLCDS-45 scores (3.57 and 3.00, respectively; Fig. 3A) and TYLCDS-90 scores (4.60 and 4.36, respectively; Fig. 3B).

**Fig. 4:**
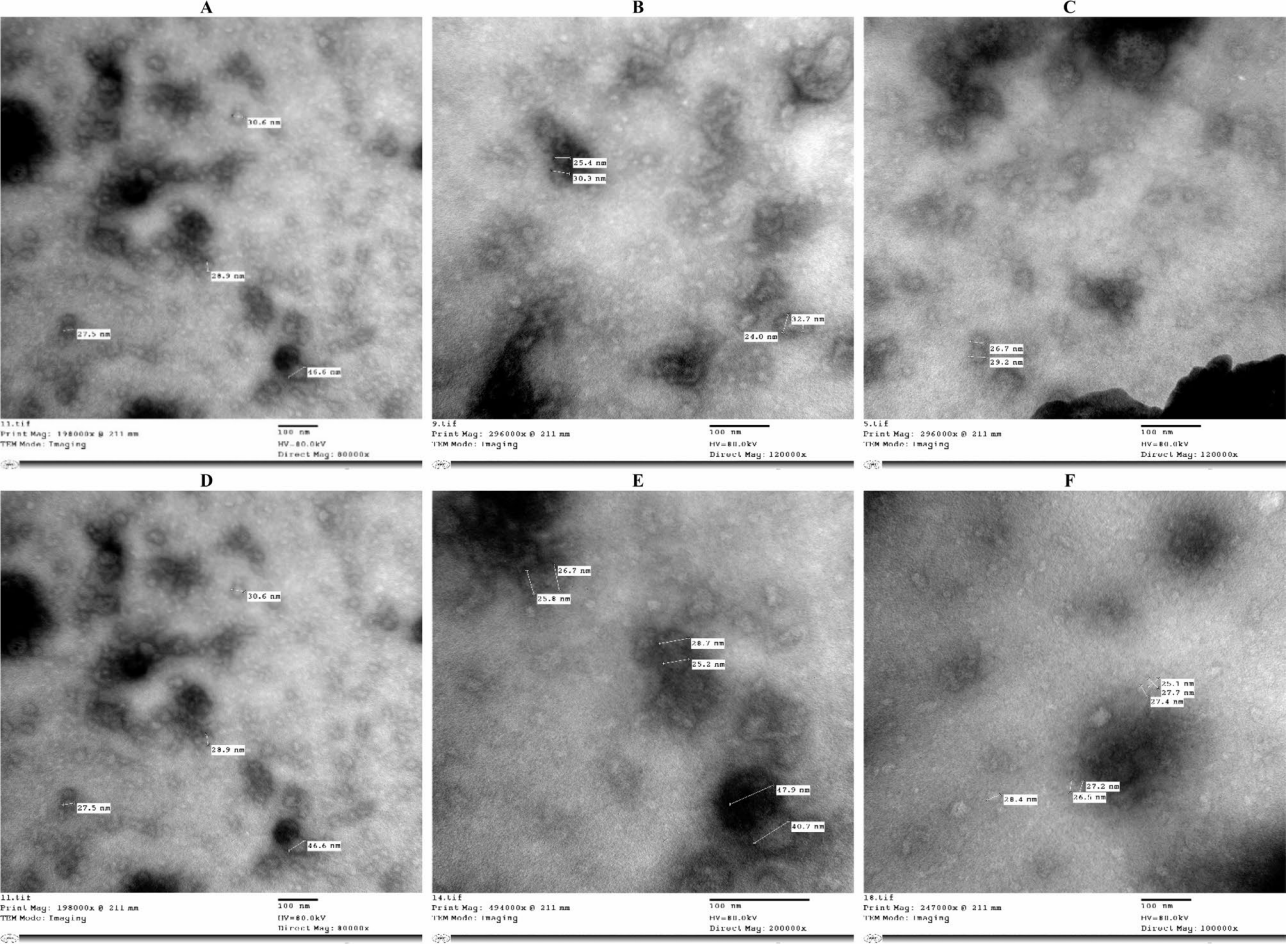
TEM images of viral particles in symptomatic ‘Castlerock’ plant and symptomless plants of some tomato lines. Symptomatic'Castlerock' plant (**A**) and symptomless plants of TL6 (**B**), TL7 (**C**), TL10 (**D**), TL15 (**E**), and TL16 (**F**). TL6: F_7_ lines of ‘65010 F_1_’; TL7, TL10, TL15, and TL16: F_7_ lines of ‘SVTD8320 F_1_’. Bar represents 100 nm

TEM was used to scan virus preparations from severe symptomatic ‘Castlerock’ plants, symptomless plants of TL6 and TL7, and mild symptomatic plants of TL10, TL15, and TL16. As shown in Fig. 4, TEM images indicated the presence of scattered twinned icosahedral particles with diameters ranging from 16.6 to 47.9 nm.

#### Vegetative traits

**Fig. 5:**
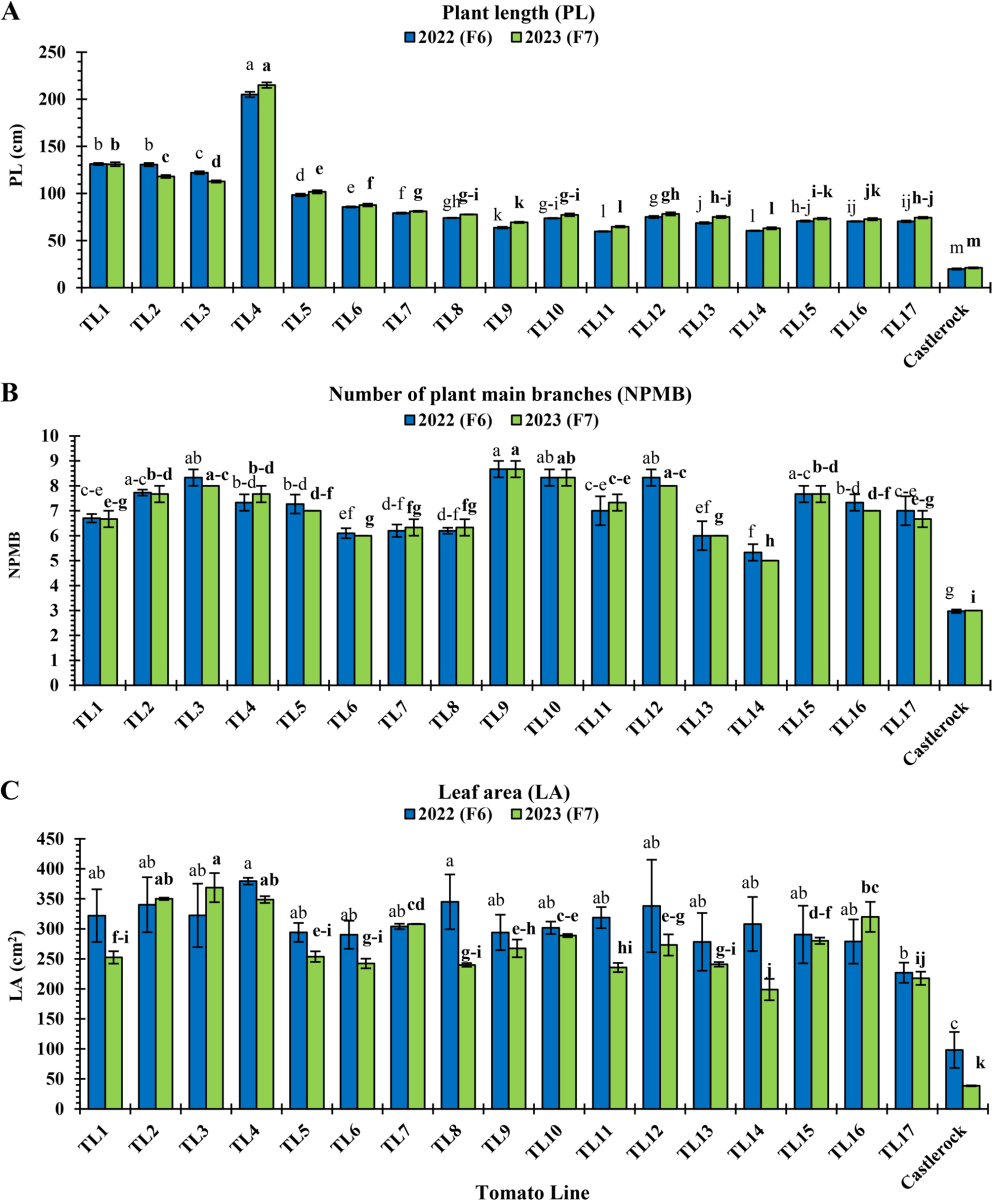
Vegetative growth performance of tomato lines and ‘Castlerock’ under natural whitefly-mediated TYLCV infection. Vegetative growth traits of plant length (**A**) number of plant main branches (**B**) and leaf area (**C**) were estimated at 90 days after transplanting. Lines were F_6_& F_7_ generations from tomato commercial F_1_ hybrids ‘Nairouz’ for TL1&TL2, ‘65010’ for TL3&TL6,‘SVTD8320’ for TL7 - TL16, and ‘Tyrmes’ for TL17. Columns with the same letter represent values that are not significantly different at the 5% probability level according to Duncan’s multiple range test. Vertical bars represent ± standard error of the means (replicates = 3)

Vegetative growth traits of the TLs, including PL, NPMB, and LA, are presented in Fig. 5. In both seasons, TL4 exhibited the tallest plants (205.0 and 215.0, respectively), followed by TL1 (131 and 118 cm, respectively) and TL2 (130.6 and 118.8 cm, respectively), with significant differences (*P* < 0.05) among them (Fig. 5A). Conversely, ‘Castlerock’ had the shortest plants in both seasons (19.77 and 21.00, respectively), followed by TL11 (59.67 and 64.70, respectively) and TL14 (60.33 and 63.00, respectively), with significant differences (*P* < 0.05) among them (Fig. 5A). In both seasons, the highest NPMB values were recorded in TL3 (8.33 and 8.00, respectively), TL9 (8.67 for both seasons), TL10 (8.33 for both seasons), and TL12 (8.33 and 8.00, respectively), with significant similarities (*P* < 0.05) among them and TL2 and TL15 in 2022 only (7.73 and 7.67, respectively) (Fig. 5B). ‘Castlerock’ had the fewest NMB in both seasons (2.97 and 3.00, respectively), followed by TL14 (5.33 and 5.00, respectively; Fig. 5B). In both seasons, the largest LA values (cm^2^) were found in TL2 (340.19 and 350.02, respectively), TL3 (322.42 and 368.64, respectively), and TL4 (379.44 and 348.76, respectively), with significant similarities among them (*P* < 0.05; Fig. 5C). ‘Castlerock’ had the smallest LA in both seasons (98.27 and 38.50, respectively), followed by TL17 (226.92 and 217.63, respectively; Fig. 5C).

#### Plant yield components

**Fig. 6:**
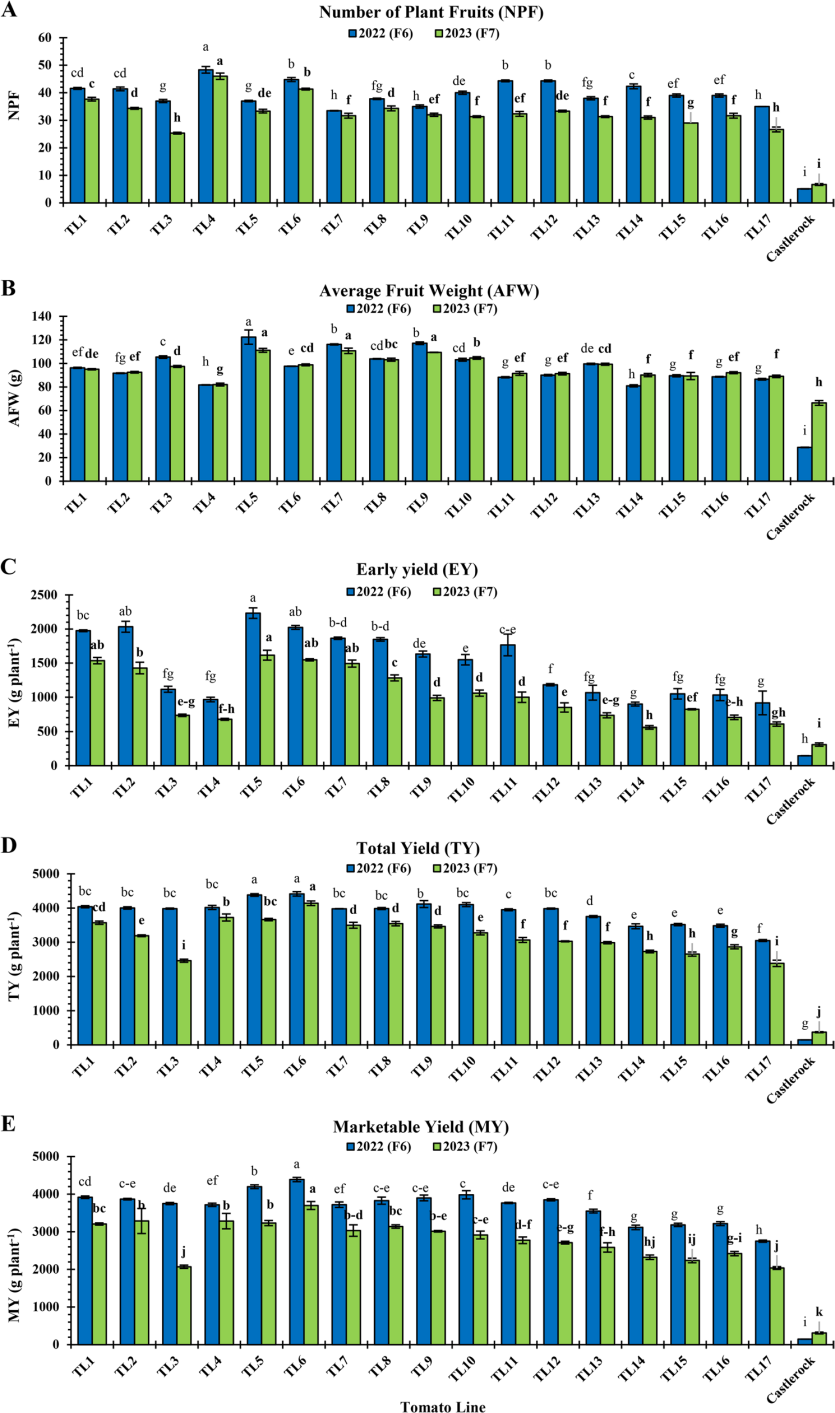
Plant yield components for tomato lines and ‘Castlerock’ under natural whitefly-mediated TYLCV infection. Plant yield components were number of plant fruits (**A**) average fruit weight (**B**) early yield (**C**) total yield (**D**) and marketable yield (**E**). Lines were F_6_& F_7_ generations from tomato commercial F_1_ hybrids ‘Nairouz’ for TL1&TL2, ‘65010’ for TL3&TL6,‘SVTD8320’ for TL7 - TL16, and ‘Tyrmes’ for TL17. Data of AFW, EY, and TY were transformed by the arcsin equation for statistical analysis. Columns with the same letter represent values that are not significantly different at the 5% probability level according to Duncan’s multiple range test. Vertical bars represent± standard error of the means (replicates = 3)

Yield components of the evaluated TLs, including NPF, AFW, EY, TY, and MY, are presented in Fig. 6. NPF ranged from 5.10 (‘Castlerock’) to 48.33 (TL4) in 2022 and 6.67 (‘Castlerock’) to 46 (TL4) in 2023 (Fig. 6A). TL4 consistently recorded the highest NPF in both seasons (48.33 and 46.00, respectively), followed by TL6 (44.80 and 41.33, respectively; Fig. 6A). ‘Castlerock’ had the lowest NPF in both seasons (5.10 and 6.67, respectively), followed by TL17 (35 and 26.67, respectively) and TL3 (37.00 and 25.33, respectively) with significant differences (*P* < 0.05) among them (Fig. 6A).

AFW (g) ranged from 28.69 (‘Castlerock’) to 122.34 (TL5) in 2022 and 66.42 (‘Castlerock’) to 111.11 (TL5) in 2023 (Fig. 6B). TL5 had the highest AFW in both seasons (122.34 and 111.11, respectively), followed by TL7 (116.10 and 110.68, respectively) and TL9 (117.15 and 109.39, respectively) with significant (*P* > 0.05) differences among them in 2022 (Fig. 6B). ‘Castlerock’ had the lowest AFW in both seasons (28.69 and 66.42, respectively), followed by TL4 (81.74 and 82.05, respectively) and TL14 (80.96 and 90.12, respectively) with significant differences (*P* < 0.05) among them (Fig. 6B).

EY (g plant^−1^) ranged from 146.33 g (‘Castlerock’) to 2233.33 (TL5) in 2022, and from 310 (‘Castlerock’) to 1616.67 (TL5) in 2023 (Fig. 6C). The highest EY in both seasons was found with TL5 (2233.33 and 1616.67, respectively) and TL6 (2023.33 and 1548.33, respectively), with significant similarities (*P* < 0.05) among them and TL2 in only 2022 (2033) and TL1 and TL7 in only 2023 (1536.7 and 1493.3, respectively; Fig. 6C). ‘Castlerock’ plants had significantly the lowest EY in both seasons (146.33 and 310, respectively), followed by TL4 (966.67 and 676.67, respectively), TL14 (900.00 and 560.00, respectively), TL16 (1033.33 and 705.00, respectively), and TL17 (916.67 and 606.67, respectively), with significant similarities (*P* < 0.05) among them (Fig. 6C).

TY (g plant^−1^) ranged from 146.33 (‘Castlerock’) to 4413.33 (TL6) in 2022, and 370 (‘Castlerock’) to 4140 (TL6) in 2023 (Fig. 6D). TL6 had the highest TY in both seasons (4413.33 and 4140, respectively), followed by TL5 (4381.67 and 3663.33, respectively), with a significant similarity (*P* < 0.05) among them in 2022 (Fig. 6D). ‘Castlerock’ recorded the lowest TY in both seasons (146.33 and 370, respectively), followed by TL17 (3050 and 2383.33, respectively; Fig. 6D).

MY (g plant^−1^) ranged from 146.33 g (‘Castlerock’) to 4388.33 g (TL6) in 2022, and 310 g (‘Castlerock’) to 3698.33 g (TL6) in 2023 (Fig. 6E). TL6 consistently achieved the highest MY in both seasons (4388.33 and 3698.33, respectively), followed by TL5 (4197.50 and 3231.67, respectively), TL1 (3918.33 and 3206.67, respectively), TL2 (3866.67 and 3286.67, respectively), TL8 (3826.67 and 3140.00, respectively), and TL9 (3900.00 and 3013.33, respectively), with significant similarities (*P* < 0.05) among them, especially in 2022 (Fig. 6E). ‘Castlerock’ had the lowest MY in both seasons (146.33 and 310, respectively), followed by TL17 (2750.00 and 2036.67, respectively; Fig. 6E).

#### Fruit quality traits

**Fig. 7:**
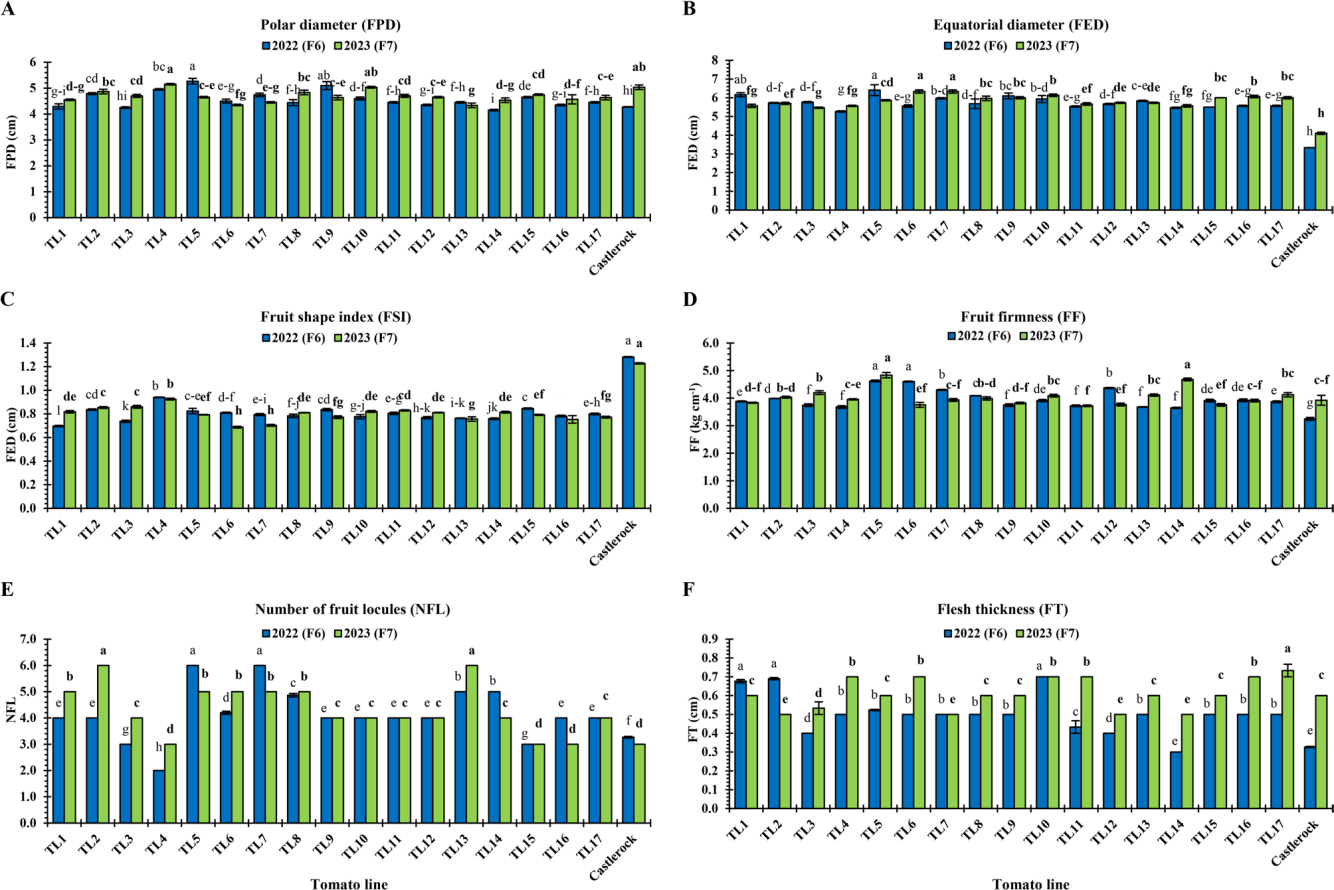
Physical fruit qualities of tomato lines and ‘Castlerock’ under natural whitefly-mediated TYLCV infection. Physical fruit qualities were polar (**A**) and equatorial (**B**) diameters, fruit shape index (**C**), fruit firmness (**D**), number of fruit locules (**E**) and flesh thickness (**F**). Lines were F_6_& F_7_ generations from tomato commercial F_1_ hybrids ‘Nairouz’ for TL1&TL2, ‘65010’ for TL3&TL6,‘SVTD8320’ for TL7 - TL16, and ‘Tyrmes’ for TL17. FSI data were transformed by the arcsin equation for statistical analysis. Columns with the same letter represent values that are not significantly different at the 5% probability level according to Duncan’s multiple range test. Vertical bars represent ± standard error of the means (replicates = 3)

Fruit physical traits including FPD, FED, FSI, FF, NFL, and FT, varied across TLs and seasons as presented in Fig. 7. FPD (cm) ranged from 4.15 (TL14) to 5.27 (TL5) in 2022, and from 4.33 (TL13) to 5.15 (TL4) in 2023 (Fig. 7A). Highest FPD values were inconsistent among both generations: TL5 and TL9 in F_6_ (2022: 5.27 and 5.10, respectively); TL4, TL10, and ‘Castlerock’ in F7 (2023: 5.15, 5.03, and 5.03, respectively; Fig. 7A). In both seasons, the lowest FPD was for TL1 (4.29 and 4.55, respectively) and TL14 (4.15 and 4.53, respectively), with significant similarities (*P* < 0.05) among them and TL12 and TL16 in 2022 (4.35 for both), and TL6, TL7, and TL13 in 2023 (4.35, 4.45, and 4.33, respectively) (Fig. 7A).

FED (cm) ranged from 3.30 (‘Castlerock’) to 6.41 (TL5) in 2022, and from 4.10 (‘Castlerock’) to 6.33 (TL6 and TL8) in 2023 (Fig. 7B). The highest FED was for TL5 and TL1 in 2022 (6.41 and 6.16, respectively), and for TL6 and TL7 in 2023 (6.33 for both; Fig. 7B). In both seasons, the lowest FED was for ‘Castlerock’ (3.30 and 4.10, respectively), followed by TL4 (5.27 and 5.57, respectively) and TL14 (5.47 and 5.57, respectively) (Fig. 7B).

FSI ranged from 0.70 (TL1) to 1.28 (‘Castlerock’) in 2022, and from 0.70 (TL7) to 1.23 (‘Castlerock’) in 2023 (Fig. 7C). According to Yeager [49], TLs had oblate fruits (FSI ranging 0.70–0.93 in 2022 and 0.69–0.93 in 2023; Fig. 7C), while ‘Castlerock’ had oval fruits (FSI being 1.28 and 1.23 in 2022 and 2023, respectively; Fig. 7C) (Fig. S3).

FF (kg cm^−2^) ranged from 3.25 (‘Castlerock’) to 4.62 (TL5) in 2022, and from 3.73 (TL11) to 4.83 (TL5) in 2023 (Fig. 7D). TL5 had the highest FF across seasons (4.62 and 4.83, respectively), with significant similarities (*P* < 0.05) with TL6 in 2022 (4.60) and TL14 in 2023 (4.70) (Fig. 7D). The lowest FF was for ‘Castlerock’ (3.25 and 3.93, respectively), TL9 (3.74 and 3.83, respectively), and TL11 (3.72 and 3,73, respectively), with significant similarities (*P* < 0.05) among them in 2023 and TL1, TL6, TL7, TL9, TL11, TL12, TL15, TL16 in only 2023 (ranging 3.73–3.90; Fig. 7D).

NFL ranged from 2 (TL4) to 6 (TL5 and TL7) in 2022, and from 3 (TL4, TL15 TL16, and ‘Castlerock’) to 6 (TL2 and TL13) in 2023 (Fig. 7E). Highest NFL values were inconsistent among both generations: TL5 and TL7 in F_6_ (2022: 6 for both); and TL2 and TL13 in F_7_ (2023: 6 for both). The lowest NFL in both seasons was in TL4 (2 and 3, respectively) and TL15 (3 for both), with significant similarities (*P* < 0.05) among them in 2023 (Fig. 7E).

FT (cm) ranged from 0.30 (TL14) to 0.70 (TL10) in 2022, and from 0.50 (TL2, TL7, TL12, and TL14) to 0.73 (TL17) in 2023 (Fig. 7F). Highest FT values were inconsistent among both generation: TL1, TL2, and TL10 in F_6_ (2022); and TL17 in F_7_ (2023) (Fig. 7F).

**Fig. 8:**
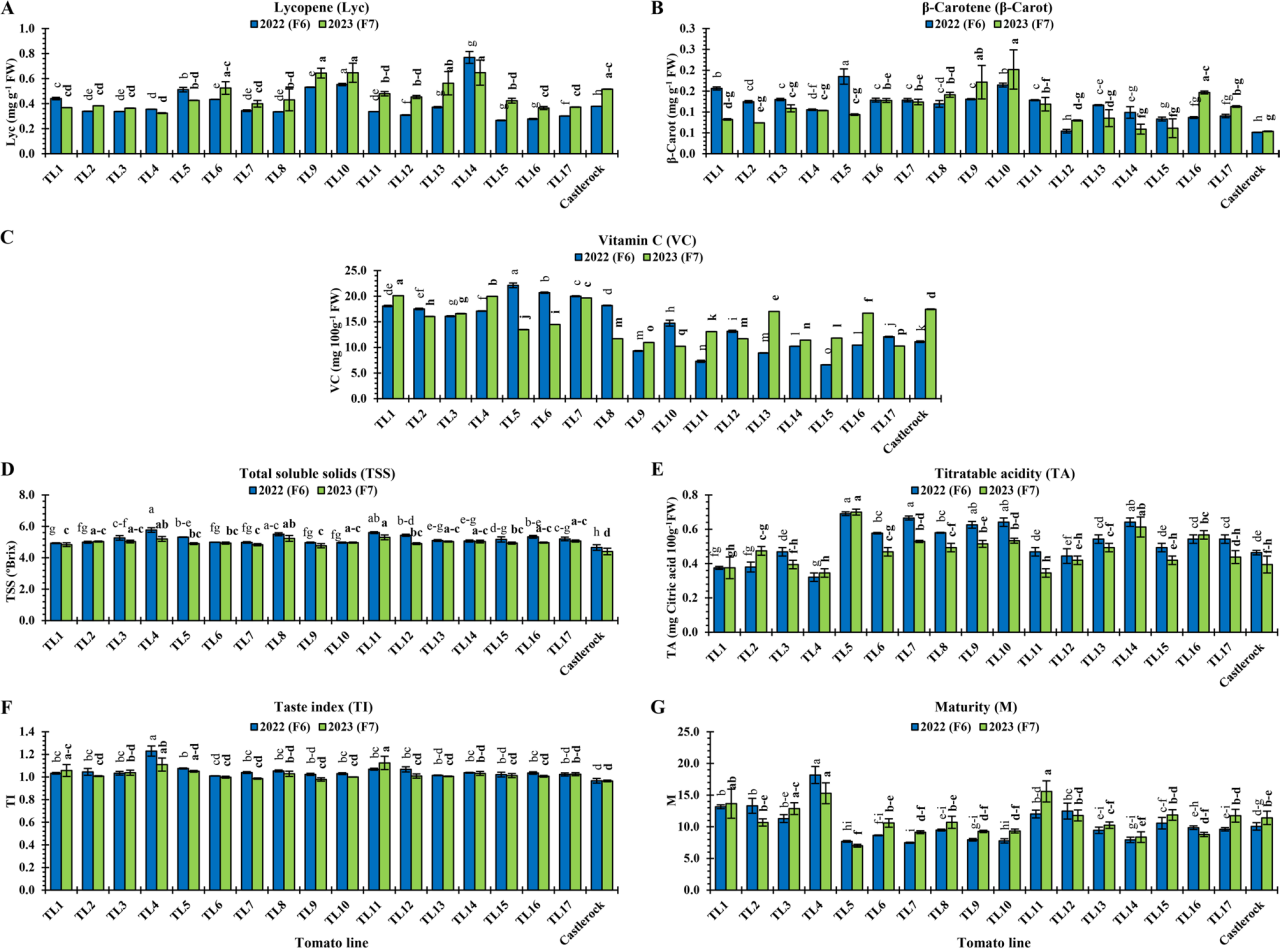
Chemical fruit qualities of tomato lines and ‘Castlerock’ under natural whitefly-mediated TYLCV infection. Chemical fruit qualities were content of lycopene (**A**) β-carotene (**B**) vitamin C (**C**) total soluble solids (**D**) and titratable acidity (**E**) as well as taste index (**F**) and maturity (**G**). Taste indices were estimated using values of TSS and titratable acidity according to Navez et al. [54]. Lines were F_6_& F_7_ generations from tomato commercial F_1_ hybrids ‘Nairouz’ for TL1&TL2, ‘65010’ for TL3&TL6,‘SVTD8320’ for TL7 - TL16, and ‘Tyrmes’ for TL17. Data of TA, TI, and M were transformed by the arcsin equation for statistical analysis. Columns with the same letter represent values that are not significantly different at the 5% probability level according to Duncan’s multiple range test. Vertical bars represent ± standard error of the means (replicates = 3)

Fruit chemical quality traits including VC, Lyc, β-Car, TSS, TA, TI, and M, of TLs are shown in Fig. 8. Fruit Lyc (mg g^−1^ FW) ranged from 0.27 (TL15) to 0.55 (TL10) in 2022, and 0.32 (TL4) to 0.65 (TL10 and TL14) in 2023 (Fig. 8A). TL10 showed the highest Lyc in both seasons (0.55 and 0.65, respectively), with significant similarities (*P* < 0.05) to TL6, TL9, TL13, TL14, and ‘Castlerock’ in only 2023 (ranging 0.52–0.65; Fig. 8A). Lowest Lyc values were inconsistent among both generations: ‘Castlerock’ in F_6_ (2022: 0.18), and TL4 in F_7_ (2023: 0.32) (Fig. 8A).

Fruit β-Car (mg g^−1^ FW) ranged from 0.051 (‘Castlerock’) to 0.185 (TL5) in 2022, and from 0.05 (‘Castlerock’) to 0.20 (TL10) in 2023 (Fig. 8B). Highest β-Car values were inconsistent among both generations: TL5 in F_6_ (2022: 0.185); and TL9, TL10, and TL16 in F_7_ (2023: 0.17, 0.20, and 0.15, respectively). In both seasons, the lowest β-Car was in ‘Castlerock’ (0.051 and 0.05, respectively) and TL12 (0.054 and 0.08, respectively), with significant similarities (*P* < 0.05) among them (Fig. 8B).

VC (mg 100 g^−1^ FW) ranged from 6.61 (TL15) to 22.12 (TL5) in 2022 and 10.22 (TL10) to 20.10 (TL1) in 2023 (Fig. 8C). Highest VC values were inconsistent among both generations: TL5, TL6, and TL7 in F_6_ (2022: 22.12, 20.69, and 19.99, respectively); TL1, TL4, and TLl7 in F_7_ (2023) Fig. 8C). The lowest VC values were also inconsistent among both generations, TL15 in F_6_ (2022: 6.61) and for TL10 in F_7_ (2023: 10.22) (Fig. 8C**).**

Fruit TSS (°Brix) ranged from 4.65 (‘Castlerock’) to 5.77 (TL4) in 2022, and from 4.40 (‘Castlerock’) to 5.30 (TL11) in 2023 (Fig. 8D). In both seasons, the highest TSS was found in TL4 (5.77 and 5.22, respectively), TL8 (5.50 and 5.23, respectively), and TL11 (5.60 and 5.30, respectively), with significant similarities (*P* < 0.05) among them, and TL2, TL3, TL10, TL13, TL14, TL16, and TL17 in 2023 (ranging 4.97–5.03). ‘Castlerock’ showed the lowest TSS in both seasons (4.65 and 4.40, respectively), followed by TL1 (4.93 and 4.80, respectively), TL7 (4.98 and 4.83, respectively), and TL6 (4.99 and 4.90, respectively) with significant differences (*P* < 0.05) among them (Fig. 8D).

Fruit TA content (mg citric acid 100 g^−1^ FW) ranged from 0.32 (TL4) to 0.69 (TL5) in 2022, and from 0.35 (TL4 and TL11) to 0.70 (TL5) in 2023 (Fig. 8E). In both seasons, the highest TA showed in TL5 (0.69 and 0.70, respectively), TL7 (0.67 and 0.53, respectively), TL9 (0.63 and 0.52, respectively), TL10 (0.64 and 0.53, respectively), and TL14 (0.64 and 0.61, respectively), with significant similarities (*P* < 0.05) among them in 2022. In both seasons, the lowest TA showed in TL4 (0.32 and 0.35, respectively) and TL1 (0.38 for both), with significant similarities (*P* < 0.05) among them and TL2 in only 2022 (0.38), and TL3, TL11, TL12, TL15, TL17, and ‘Castlerock’ in only 2023 (ranging 0.35–0.44) (Fig. 8E).

Fruit TI ranged from 0.97 (‘Castlerock’) to 1.23 (TL4) in 2022, and from 0.97 (‘Castlerock’) to 1.12 (TL11) in 2023 (Fig. 8F). In both seasons, the highest TI was showed with TL4 (1.23 and 1.11), with significant similarities (*P* < 0.05) among them and TL1, TL5, and TL11 in only 2023 (1.06, 1.05, and 1.12, respectively). In both seasons, the lowest TI was shown in TL6 (1.01 and 1.00), TL9 (1.02 and 0.98), TL13 (1.01 for both), TL15 (1.02 and 1.01, respectively), TL17 (1.02 and 1.03, respectively), and ‘Castlerock’ (0.97 for both) (Fig. 8F).

Fruit M ranged from 7.48 (TL7) to 18.18 (TL4) in 2022, and from 7.01 (TL5) to 15.59 (TL11) in 2023 (Fig. 8G). The highest M was in TL4 in both seasons (18.18 and 15.29, respectively) with significant similarities (*P* < 0.05) to TL1, TL3, and TL11 in 2023 (13.65, 12.87, and 15.59, respectively). In both seasons, the lowest M was in TL5 (7.70 and 7.01, respectively), TL7 (7.48 and 9.14, respectively), TL9 (7.95 and 9.27, respectively), TL10 (7.77 and 9.33, respectively), TL13 (9.44 and 10.2, respectively), and TL14 (7.93 and 8.37, respectively) (Fig. 8G).

### Genetic parameters

Table 2 presents the estimated variability components (*δ*^*2*^_*g*_, *δ*^*2*^_*gy*_, *δ*^*2*^_*e*_, *δ*^*2*^_*ph*_, *PCV*, *ECV*, and *GCV*) and *h*^*2*^_*b*_ for 27 phenotypic traits related to TYLCD-resistance, vegetative growth, yield components, and fruit quality in tomato lines. PCV% values ranged from 4.14 (TI) to 45.51 (PL). High PCV% (20.99–45.51%) was found for TYLCD-S-45, TYLCD-S-90, PL, LA, NPF, EY, TY, MY, NFL, VC, Lyc, B-Car, and M. Moderate PCV% (11.94–19.58%) was observed for NPB, Chlor-a, Chlor-b, T-Chlor, T-Car, AFW, FSI, FT, and TA. Low PCV% (4.14–9.36%) was estimated for PD, ED, FF, TSS, and TI (Table 2). GCV ranged from 2.45% (FPD) to 45.41% (PL). High GCV% (20.79–45.41%) was found in TYLCD-S-45, TYLCD-S-90, PL, LA, NPF, EY, TY, MY, Lyc, and M. Moderate GCV% (13.40–19.52%) was observed for NPB, AFW, FSI, NFL, VC, and TA. Low GCV% (2.61–9.93%) was found for Chlor-a, Chlor-b, T-Chlor, T-Car, PD, ED, FF, FT, TSS, and TI (Table 2). ECV ranged from 0.75% (NFL) to 46.69% (T-Carot). High ECV% (28.23–46.69%) was found with chlor-a, chlor-b, t-chlor, t-carot, and β-carot. Moderate ECV% (12.36–16.04%) was identified for TYLCDS-45, TYLCDS-90, LA, Lyc, and M (Table 2). Low ECV% (0.75–9.40%) was observed for PL, NPB, NPF, AFW, EY, TY, MY, PD, ED, FSI, FF, NFL, FT, TSS, VC, TA, and TI (Table 2).

The *h*^*2*^_*b*_ ranged from 18.41% (FF) to 99.57% (PL) (Table 2). Low *h*^*2*^_*b*_ was found for FF (18.41); moderate (30.08–52.82%) for Chlor-a, Chlor-b, T-Chlor, T-Car, FPD, FT, and VC; while high (75.00–99.57%) for the other estimated traits (Table 2).

### Genotypic and phenotypic correlations

Table 3 presents phenotypic (*r*_*ph*_) and genotypic (*r*_*g*_) correlation coefficients for all study traits. TYLCDS-45 showed significant positive genotypic and phenotypic correlations with FSI (*r*_*ph*_: 0.562 and *r*_*g*_: 0.617), and positive genotypic correlations with Chlor-b (*r*_*g*_: 0.495) and T-Car (*r*_*g*_: 0.501). However, it had significant negative correlations (both *r*_g_ and *r*_*ph*_) with each of PL, LA, EY, TY, MY, NPF, AFW, FED, FF, and VC, with *r*_*g*_ values ranging from − 0.580 to −0.792 and *r*_*ph*_ values from − 0.616 to −1.000 (Table 3**)**. TYLCDS-90 had a significant positive phenotypic and genotypic correlation with only FSI (*r*_*ph*_: 0.745 and *r*_*g*_: 0.808), but significant negative genotypic and phenotypic correlations with PL, LA, EY, TY, MY, NPF, AFW, FED, FF, NFL, and VC, with *r*_*g*_ values ranging from − 0.451 to −0.909 and *r*_*ph*_ values from − 0.579 to −1.000, and a negative genotypic correlation with FFT (*r*_*g*_: 0.450).

**Table 3:**
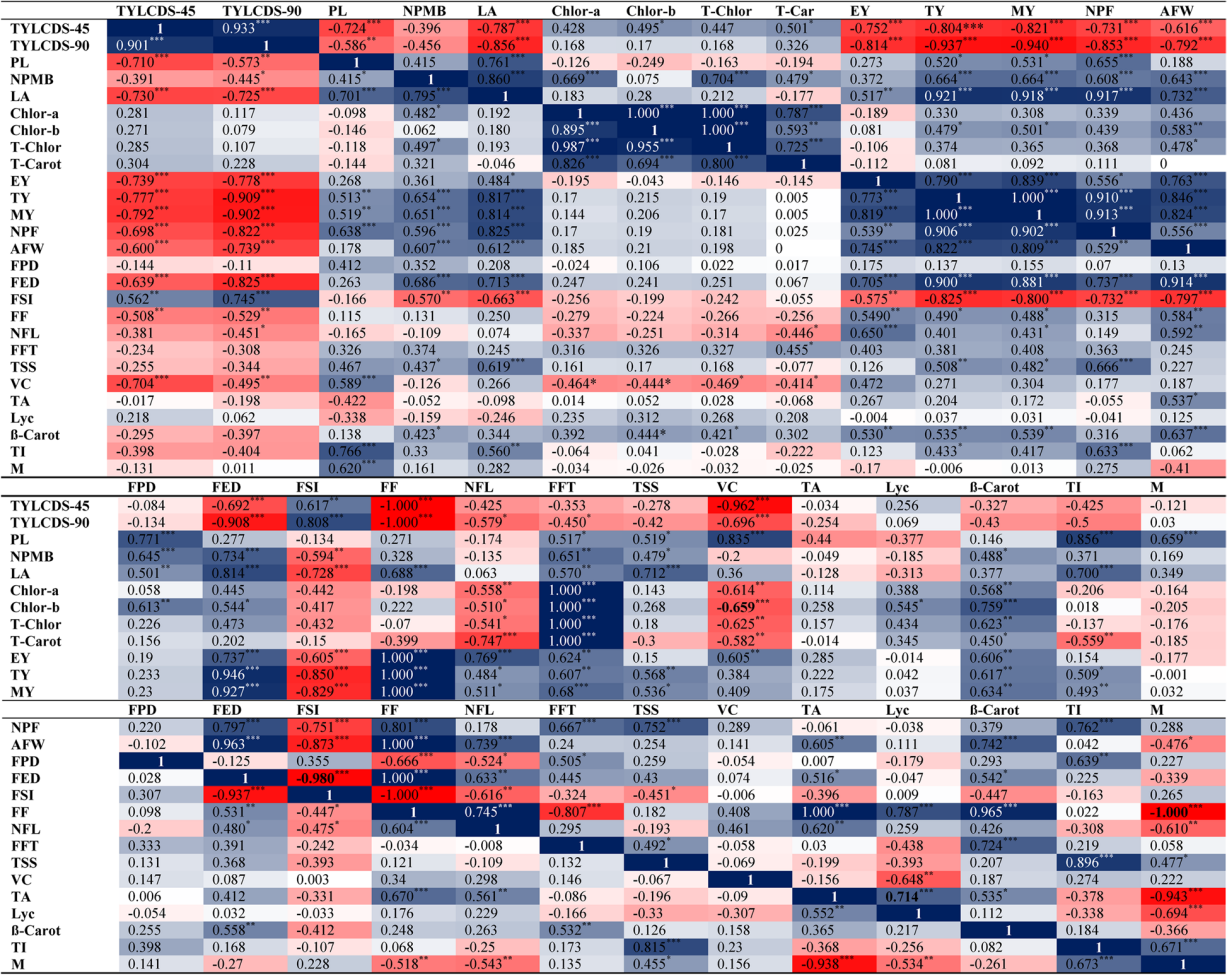
Genotypic (above diagonal) and phenotypic (below diagonal) correlations between 27 traits of tomato lines

TY showed significant negative phenotypic and genotypic correlations with either TYLCDS-45 (*r*_*ph*_: −0.777 and *r*_*g*_: −0.804), TYLCDS-90 (*r*_*ph*_: −0.909 and *r*_*g*_: −0.937), and FSI (*r*_*ph*_: −0.825 and *r*_*g*_: −0.850) (Table 3). Conversely, TY had significant positive phenotypic and genotypic correlations with either PL, NPMB, LA, EY, MY, NPF, AFW, FED, FF, TSS, β-Car, and TI (*r*_*ph*_: 0.490–1.000; *r*_*g*_: 0.520–1.000), as well as positive genotypic correlations with Chlor-b (0.479), NFL (0.484), and FFT (0.607) (Table 3).

AFW showed significant negative phenotypic and genotypic correlations with TYLCDS-45, TYLCDS-90, FSI (*r*_*ph*_: −0.600 to −0.873 and *r*_*g*_: −0.616 to −0.797; Table 3), but positive phenotypic and genotypic correlations with NPB, LA, EY, MY, NPF, FED, FF, NFL, TA, and β-Car (*r*_*ph*_: 0.529 to 1.000, rg: 0.537 to 0.914; Table 3).

TSS showed significant positive phenotypic and genotypic correlations with PL, NPMB, LA, TY, MY, NPF, TI, and M with (*r*_*ph*_: 0.473 to 0.896 and *r*_*g*_: 0.455 to 0.815); positive genotypic correlation with FFT (*r*_*g*_: 0.492); and a significant negative genotypic correlation with FSI (*r*_*g*_: −0.451) (Table 3).

FF had significant negative phenotypic and genotypic correlations with each of TYLCDS-45, TYLCDS-90, FSI, and M (*r*_*ph*_: −0.508 to −1.000 and *r*_*g*_: −0.518 to −1.000); a significant negative genotypic correlation with FPD (*r*_*g*_: −0.666); and a significant negative phenotypic correlation with FFT (*r*_*ph*_: −0.807). FF showed significant positive phenotypic and genotypic correlations with EY, TY, MY, AFW, FED, NFL, and TA (*r*_*ph*_: 0.488–1.000; *r*_*g*_: 0.604–1.000; Table 3); and significant positive genotypic correlations with LA and NPF (*r*_*g*_: 0.688 and 0.801, respectively) (Table 3).

### Multivariate analysis

**Fig. 9:**
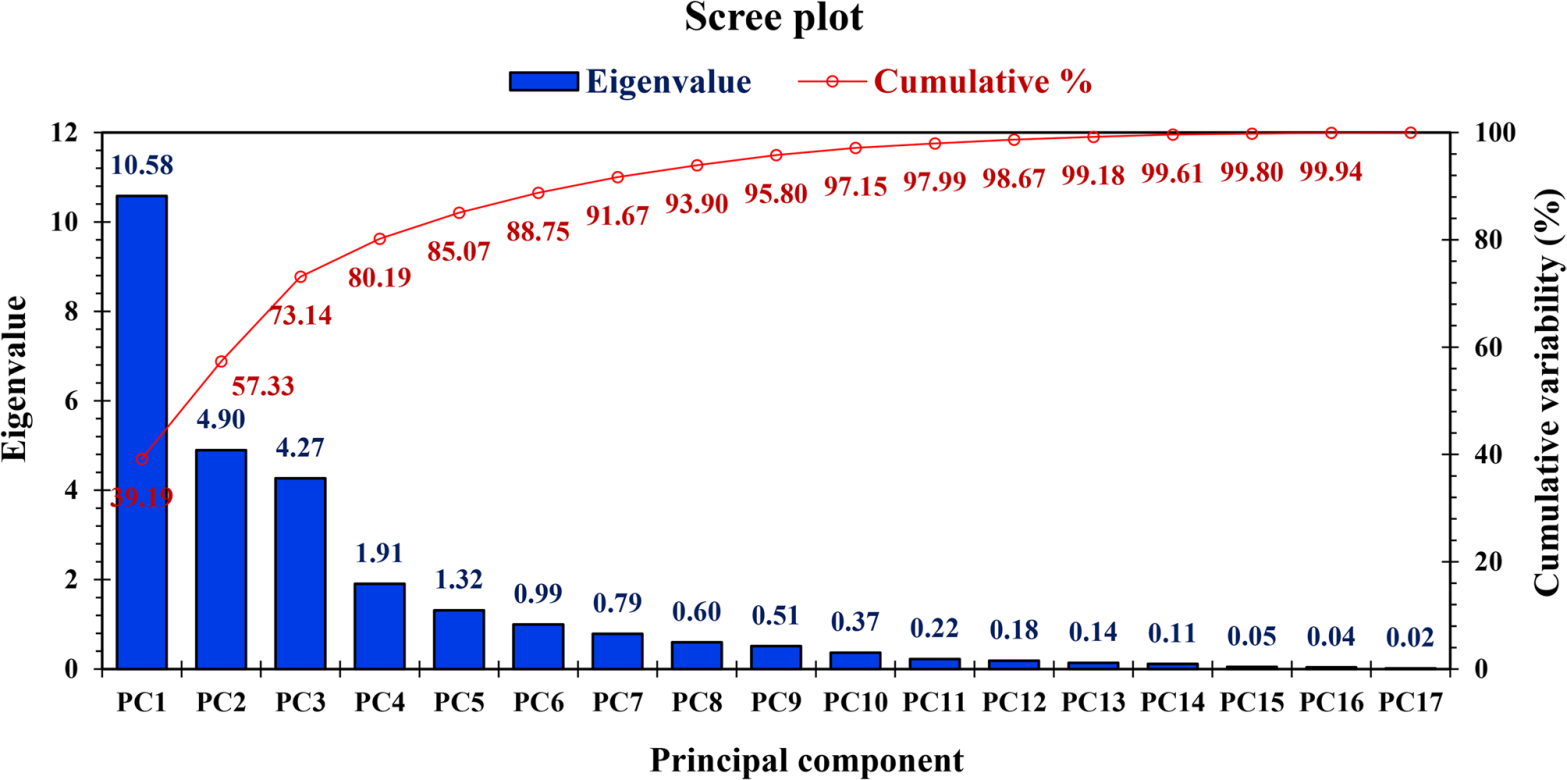
Scree plot of 17 principal components for 27 traits of tomato lines and ‘Castlerock’

PCA was conducted using 27 morphological traits of TLs grown under natural TYLCD infection during the fall seasons of 2022 and 2023. A total of 17 PCs were identified, with eigenvalues ranging from 10.58 to 0.02 (Fig. 9). **T**he first five PCs accounted for the majority of trait variation (Table 4). PC1 explained 39.19% of the total variance (eigenvalue = 10.58; Fig. 9), with negative loadings for TYLCD-S-45 (−0.826), TYLCD-S-90 (−0.908), and FSI (−0.794), and positive loadings for NPMB (0.696), LA (0.855), T-Chlor (0.566), EY (0.781), TY (0.979), NPF (0.872), AFW (0.831), FED (0.899), TSS (0.506), and β-Carot (0.656) (Table 4). PC2 accounted for 18.14% of the variance (eigenvalue = 4.90; Fig. 9), with positive loadings for PL (0.630), TI (0654), and M (0.942), and negative loadings for FF (−0.538), NFL (−0.738), and TA (−0.872) (Table 4). PC3 explained 15.80% of the variance (eigenvalue = 4.27; Fig. 9), and was mainly defined by positive loadings for Lyc (0.373), Chlor-a (0.931), Chlor-b (0.890), and T-Carot (0.865), and a negative loading for VC (−0.645) (Table 4). PC4 contributed 7.06% of the total variance (eigenvalue = 1.91), and was defined by a negative loading for FT (−0.469; Table 4). PC5 explained 4.87% of the variance (eigenvalue = 1.32; Fig. 9), and showed a positive loading for FPD (0.629; Table 4).

**Table 4:** Component loading values of the first five principal components for 27 traits of tomato lines and ‘Castlerock’

** Trait**^z^	**PC1**	**PC2**	**PC3**	**PC4**	**PC5**
TYLCDS-45	**-0.826**	-0.040	0.454	-0.138	0.158
TYLCDS-90	**-0.908**	0.098	0.266	0.027	0.079
PL	0.596	**0.630**	-0.327	0.200	0.075
NPB	**0.696**	0.259	0.411	-0.095	0.073
LA	**0.855**	0.303	-0.013	-0.196	0.024
Chlor-a	0.146	0.151	**0.931**	-0.062	-0.074
Chlor-b	0.182	0.114	**0.890**	-0.010	0.080
T-Chlor	**0.566**	0.447	0.239	0.416	-0.086
T-Carot	-0.004	0.162	**0.865**	0.216	-0.216
EY	**0.781**	-0.305	-0.198	0.242	-0.145
TY	**0.979**	-0.050	0.036	-0.100	-0.005
MY	**0.974**	-0.044	0.017	-0.050	-0.034
NPF	**0.872**	0.239	0.005	-0.266	0.002
AFW	**0.831**	-0.429	0.121	-0.010	0.038
FPD	0.215	0.284	0.020	0.548	**0.629**
FED	**0.899**	-0.285	0.167	-0.178	-0.076
FSI	**-0.794**	0.287	-0.152	0.420	0.249
FF	0.519	**-0.538**	-0.282	0.065	0.300
NFL	0.378	**-0.738**	-0.324	-0.017	-0.105
FT	0.447	0.209	0.328	**0.469**	-0.232
TSS	**0.506**	0.464	0.000	-0.491	0.323
VC	0.363	0.070	**-0.645**	0.510	-0.196
TA	0.179	**-0.872**	0.135	0.043	0.347
Lyc	-0.057	-0.515	**0.373**	0.088	0.312
ß-Carot	**0.656**	-0.223	0.441	0.403	0.049
TI	0.461	**0.654**	-0.219	-0.140	0.434
M	0.025	**0.942**	-0.189	-0.086	-0.121

**Fig. 10:**
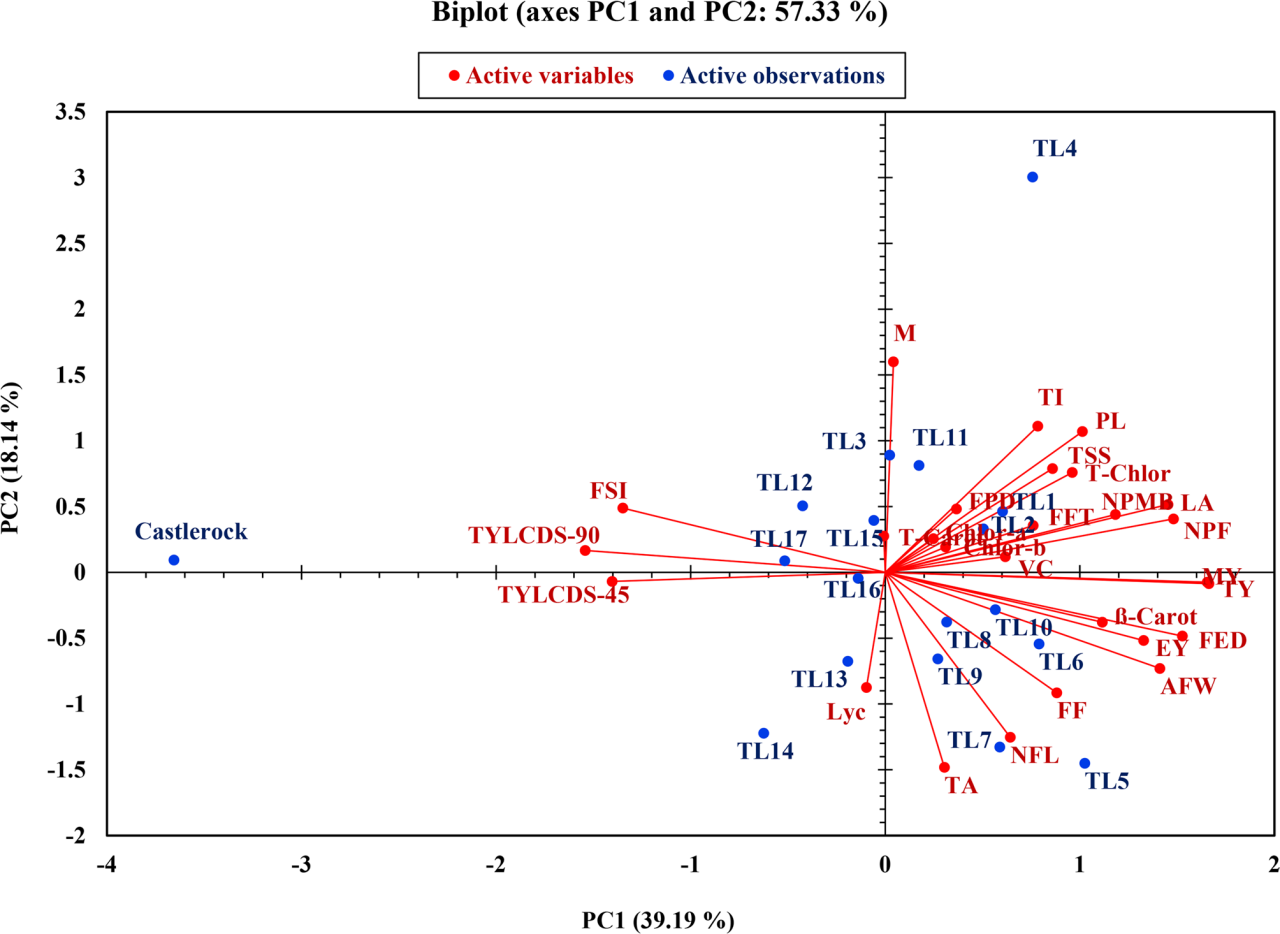
Biplot among the first two principal components for 27 traits of tomato lines and ‘Castlerock’. Tomato lines were the selected F_7_ lines from tomato commercial F_1_ hybrids ‘Nairouz’ for TL1&TL2, ‘65010’ for TL3&TL6,‘SVTD8320’ for TL7 - TL16, and ‘Tyrmes’ for TL17. Traits were related to TYLCD severity at 45 and 90DAT (TYLCDS-45 and TYLCS-90), vegetative growth traits (PL: plant length, NPMB: number of plant main branches, LA: leaf area, and Chlor-a, Chlor-b, T-chlor, and T-Carot: leaf content of chlorophyll a, b, and total, and total carotenoids, respectively), plant yield components (NPF: number of plant fruits, AFW: average fruit weight, and EY, TY, and MY: early, total, and marketable yield, respectively), and fruit quality (PD and ED: polar and equatorial diameter, FSI: fruit shape index, FF: fruit firmness, NFL: number of fruit locus, FFT: fruit flesh thickness; TSS: total soluble solids content, VC: vitamin C content, TA: titratable acidity content, Lyc: lycopene content, ß-Carot: ß-carotene content, TI: taste index, and M: maturity index)

Figure 10 shows the PCA-biplot illustrating the distribution of tomato lines and trait vectors. The biplot revealed four distinct trait groups. TYLCDS-90 and FSI vectors were grouped in a unique quadrant. TYLCDS-45 appeared in a separate quadrant alongside Lyc, but remained directionally aligned with TYLCDS-90 and FSI (Fig. 10). The remaining trait vectors were distributed across two opposite quadrants, with closely grouped and similarly oriented arrows (Fig. 10). Chlor-a, Chlor-b, T-Carot, and FPD had short arrows (Fig. 10). Tomato lines were distributed across all biplot quadrants (Fig. 10). ‘Castlerock’, TL3, TL12, TL15, and TL17 were located in a unique quadrant. ‘Castlerock’ was located at the far of TYLCDS-45, TYLCDS-90, and FSI vectors, while the other lines were located near the origin, aligning with the T-Carot vector (Fig. 10). TL1, TL4, and TL11 appeared in a separate quadrant. Notably, TL4 was at the extremities of the PL, LA, NPMB, Chlor-a, Chlor-b, T-Chlor, T-Carot, NPF, VC, TSS, M, and TI vectors (Fig. 10). TL5 to TL10 were grouped in another quadrant, with TL5 and TL7 located at the extremities of EY, TY, MY, AFW, FED, FF, NFL, TA, and β-Carot vectors (Fig. 10). TL13, TL14, and TL16 were positioned in a different quadrant, with TL13 and TL14 located at the extremes of the Lyc vector; while TL16 was near the origin (Fig. 10).

**Fig. 11:**
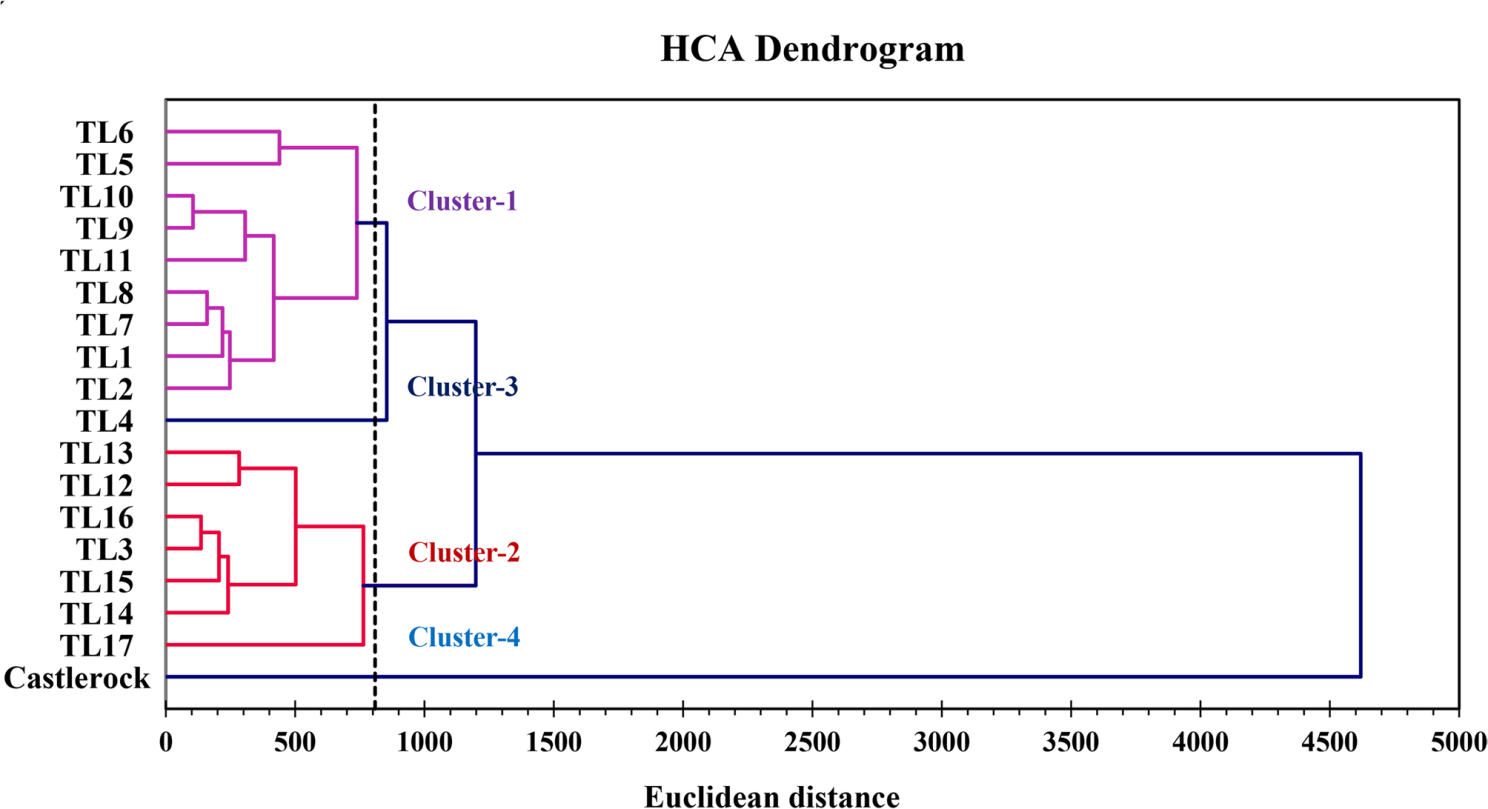
Dendrogram of hierarchical cluster analysis using Euclidean distances for tomato lines and ‘Castlerock’. Tomato lines were the selected F_7_ lines from tomato commercial F_1_ hybrids ‘Nairouz’ for TL1&TL2, ‘65010’ for TL3&TL6,‘SVTD8320’ for TL7-TL16, and ‘Tyrmes’ for TL17

HCA divided TLs into four clusters based on their estimated traits, as illustrated in Fig. 11. Cluster 1 had nine TLs, separated into two groups: TL5 and TL6 formed the first, while TL1, TL2, TL7-TL10, and TL11 formed the second (Fig. 11). This cluster exhibited the lowest M; moderate levels of TYLCD-S-45, TYLCD-S-90, PL, NPMB, LA, Chlor-a, T-Chlor, T-Carot, TY, NPF, FPD, FSI, FT, TSS, VC, and TI; and the highest values for Chlor-b, EY, MY, AFW, FED, FF, NFL, TA, Lyc, and β-Car (Table 5). Cluster 2 included 7 lines, divided into two groups: TL3 and TL12-TL16 formed the first, and TL17 formed the second (Fig. 11). This cluster showed low levels of FPD, FSI, and VC; high levels of Chlor-a and T-Carot; and moderate values for the remaining traits (Table 5). Cluster-3 included only TL4 (Fig. 5) and exhibited low levels of TYLCD-S-45, TYLCD-S-90, T-Carot, NFL, TA, and Lyc; moderate levels of Chlor-a, Chlor-b, MY, AFW, FED, FSI, FF, and β-Carot; and high levels of PL, NPMB, LA, T-Chlor, TY, NPF, FPD, FT, TSS, VC, TI, and M (Table 5). Cluster-4 included only ‘Castlerock’ (Fig. 11) and was characterized by low levels of PL, NPMB, LA, Chlor-a, Chlor-b, T-Chlor, EY, TY, MY, AFW, NPF, FED, FF, FT, TSS, β-Car, and TI; moderate levels of T-Carot, FPD, NFL, VC, TA, and M; and high levels of TYLCD-S-45, TYLCD-S-90, FSI, and Lyc (Table 5). Cluster variance ranged from 0 (Clusters 3&4) to 155122.784 (Cluster-1; Table 5). The average distance to the centroid ranged from 800.714 (Clusters 1&3) to 5073.774 (Clusters 1&4; Table 5). The minimum, average, and maximum distances to the centroid were all zero for Cluster 3 and Cluster 4, as each contained only a single genotype (Table 5).

**Table 5:**
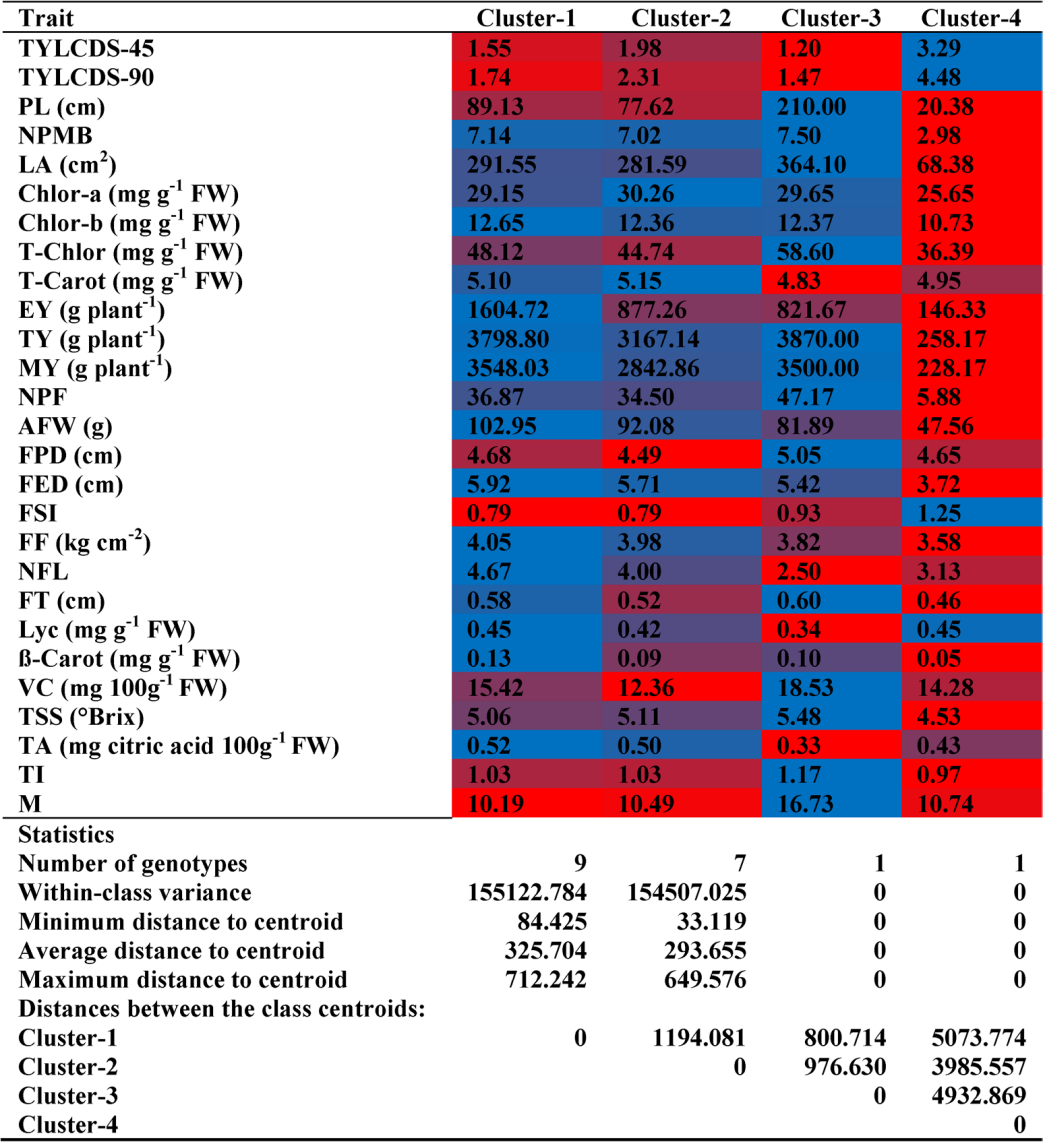
Clusters performance and statistics of clusters for tomato lines and ‘Castlerock’ based on 27 morphological traits

## Discussion

Exploring genetic diversity is essential for developing new tomato genotypes with desirable traits to improve production [10, 12]. Therefore, this study assessed genetic variability among improved tomato lines and identified *Ty* resistance genes to select elite lines with multiple resistance genes. The *Ty-1* and *Ty-3* genes are allelic or closely linked to the same gene, and are often known as *Ty-1/3* [18, 19]. To detect this gene, the TY-1/3K-SCAR marker was employed, amplifying a 102 bp fragment in susceptible genotypes and a 114 bp fragment in resistant genotypes [40]. All TLs carried the *Ty-1*/*3* resistance gene except for TL3, TL4, TL5, and TL8 (Fig. 2A). To further confirm the presence of the *Ty-*1 allele, CAPS markers TY-1 and REX-1 were employed. The REX-1 primer pair amplified a 570 bp fragment for the *ty-1* allele and a 750 bp fragment for the *Ty-1* allele [40], while the TY-1 primer pair amplified a 398 bp fragment for the *ty-1* allele and a 300 bp fragment for the *Ty-1* allele [42]. Both markers confirmed the *Ty-1* resistance allele in 12 of the 17 TLs (Fig. 2B&C). To distinguish *Ty-3*, *Ty-3a*, and *Ty-3b* alleles, the P6–25-SCAR and FER-G8-CAPS markers were used (Fig. 2D&E). The P6–25-SCAR primer pair amplified: 350 bp for *ty-3*, 450 bp for *Ty-3*, 630 bp for *T-3a*, and 660 bp for *Ty-3b* [43]. The FER-G8 marker, after digestion with Taq-1, amplified 200 bp for *ty-3* and 300 bp for *Ty-3a* [20]. *Ty-3* and *Ty-3a* alleles were detected in 12 out of 17 TLs, with TL2 and TL17 having *Ty-3*, while TL6-TL7 and TL9-TL16 carrying *Ty-3a* (Fig. 2D&E). Interestingly, TL1 did not show amplification with the TY-1/3K marker, suggesting no detectable *Ty1-3* gene. However, both TY-1 and REX-1 markers confirmed the presence of the *Ty-1*, while both FER-G8 and P6-25 markers did not detect the *Ty-3* allele. This indicates that TL1 likely carries a homozygous *Ty-1*/*Ty-1* genotype.

The TG0302-SCAR primer pair produced a 450 bp fragment for the *ty-2* allele and a 600 bp amplicon for the *Ty-2* resistant allele after *Taq-1* digestion [44]. TL2, TL7, TL9, TL10, TL13, and TL15 were homozygous for the *Ty-2* allele (Fig. 2F). The C2_At4g17300-CAPS primer pair, after digesting by *Afll*, amplified a 200 bp fragment for the *ty-4* allele and a 300 bp fragment for the *Ty-4* allele [24]. All TLs carried *Ty-4*/*Ty-4*, except TL1, TL5, and TL6 (Fig. 1G). The SINACI-CAPS primer pair, after digestion by *Taq-1*, amplified a 350 bp fragment for the *ty-5* allele and a 425 bp fragment for the *Ty-5* allele [25]. TL6, TL7, TL12, and TL13 carried *ty-5*/*ty-5* (Fig. 2H). The UF_10.61192 primer pair, after digestion by *BssHII*, amplified a 630 bp fragment for the *Ty-6* allele and a 600 bp fragment for the *ty-6* allele [27, 45]. TL2, TL4, TL8-TL10, and TL13-TL16 had the *Ty-6*/*Ty-6* (Fig. 2I).

‘Castlerock’ showed no detectable resistance genes in this study, as no bands were amplified with the specific molecular markers used for the *Ty* genes (Fig. 2). Most TLs carried multiple homozygous resistance genes (> 2), except TL3, which carried only *Ty-4/Ty-4*; and TL5, in which no resistance genes were detected. TL13 harbored all resistance genes identified. The combination of *Ty-1*/*Ty-3a*, *Ty-2*/*Ty-2*, *Ty-4*/*Ty-4*, and *Ty-6*/*Ty-6* was present in TL2, TL9, TL10, and TL15, with *Ty-3* being present in TL2. TL7 had *Ty-1*/*Ty-3*, *Ty-2*/*Ty-2*, *Ty-4*/*Ty-4*, and *ty-5*/*ty-5* genes (Fig. 2). *Ty-1*/*Ty-3a*, *Ty-4*/*Ty-4*, and *Ty-6*/*Ty-6* were found in TL14 and TL16. TL12 had *Ty-1*/*Ty-3a*, *Ty-4*/*Ty-4*, and *ty-5*/*ty-5*. Only two genes were detected in TL1 (*Ty-1/Ty-1* and *Ty-4*/*Ty-4*), TL6 (*Ty1*/*Ty-3a* & *ty-5*/*ty-5*), TL11 (*Ty-1*/*Ty-3a* & *Ty-4*/*Ty-4*), TL17 (*Ty-1*/*Ty-3* & *Ty-4*/*Ty-4*), TL4 and TL8 (*Ty-4*/*Ty-4* & *Ty-6*/*Ty-6*). These TLs were derived from commercial F_1_ hybrids known to carry multiple *Ty* resistance genes [12]. TL1 and TL2 were developed from ‘Nairouz F_1_’, which had the genes *Ty-1*/*Ty-3*, *Ty-2*/*Ty-2*, and *Ty-4*/*ty-4* [12]. TL3-TL6 originated from ‘65010 F_1_’, which had the *Ty-1*/*Ty-3a* and *Ty-4*/*ty-4*. TL7-TL16 were derived from ‘SVTD8320 F_1_,’ which had the *Ty-1*/*Ty-3a*, *Ty-2*/*Ty-2*, *Ty-4*/*ty-4*, and *Ty-5*/*ty-5*. TL17 originated from ‘Tyrmes F_1_’, which carried*Ty-1*/*Ty-3a*, *Ty-2*/*Ty-2*, *Ty-4*/*ty-4*, and *Ty-5*/*ty-5*.

Morphological traits are widely used in plant breeding to assess genetic variability due to their ease of scoring, quick analysis, and cost-effectiveness [10]. In this study, genetic diversity among TLs was assessed using ANOVA and genetic parameters of PCV, GCV, *r*_*ph*_, *r*_*g*_, and *h*^*2*^_*b*_ [10, 12, 64]. Combined ANOVA revealed significant year-to-year differences (*MS*_*y*_) in 12 of 27 traits, including vegetative growth (PL), productivity (NPF, EY, TY, and MY), and fruit quality (PD, NFL, FT, TSS, VC, TA, and Lyc) (Table 2). These differences are likely due to climatic differences between seasons, with higher temperatures and lower precipitation in the second season (Fig. 1**)**, which may have limited nutrient uptake and reduced productivity [65, 66]. According to Maršic et al. [67], tomato fruit quality declines under high temperatures and low precipitation, reducing the accumulation of vitamin C, lycopene, and β-carotene [68, 69]. Conversely, traits of foliar photosynthesis pigment content, NPB, LA, AFW, ED, FSI, FF, β-Car, TI, M, TYLCD-S, and TYLCD-I showed significant similarities across seasons (Table 2), suggesting low environmental influence and genetic stability in the evaluated lines [70]. Genotypes across seasons accounted for the majority of the total variance (43.42–99.29%) and significantly influenced most traits, except for leaf photosynthesis pigment content (Table 2). This indicates significant phenotypic variation among TLs, providing opportunities for improving TYLCD tolerance, vegetative growth, and productivity through phenotypic selection [9].

Although the susceptible ‘Castlerock’ showed visible yellowing symptoms, its leaf photosynthetic pigment content did not significantly differ from that of the tolerant TLs (Table 2). This may be due to limitations in the mass-based measurement method, which can overlook structural changes, such as curling or thickening, that affect cell density and skew pigment estimates. Similar differences have been reported in virus-infected tomato plants [71]. For more accurate assessments, future studies should consider using SPAD meters, area-based spectrophotometry, or microscopy, as these better reflect true chlorophyll concentrations and account for virus-induced changes in leaf morphology.

The *MS*_*g*_ values were higher than the *MS*_*gy*_ values by a factor ranging from 1.23 to 231.56 times (Table 2). Therefore, emphasis was placed on determining genotype-specific effects and comparing their means for the estimated traits [56].

TYLCD symptoms, including yellowing and curling of leaves, typically appear 15–21 days after infection in susceptible plants and worsen with age, causing severe reductions in growth and fruit production [2]. In this study, TYLCD symptom severity was assessed for 45 and 90 DAT under natural whitefly-mediated TYLCV infection. The susceptible ‘Castlerock’ plants, lacking *Ty* genes (Fig. 2), exhibited severe symptoms by 45DAT, fully developing by 90 DAT (Fig. 3). This high infection severity led to reduced vegetative growth, delayed development, and poor fruit production, resulting in fewer, smaller, and less marketable fruits, and ultimately, significantly lower yields (Figs. 5 and 6).

Most TLs carried multiple *Ty* genes (Fig. 2) and exhibited mild or no TYLCD symptoms, with delayed onset even under high inoculum pressure (Fig. 3). Despite being symptomless, viral replication was confirmed via TEM imaging (Fig. 4), which revealed scattered, twinned icosahedral particles ranging from 16.6 to 47.9 nm in diameter, broader than the typical 18–36 nm reported by Gafni [72] and Czosnek [8]. This size variation may result from particle aggregation, sample preparation artifacts, or the presence of defective virions. Further high-resolution or complementary analyses are recommended to better understand their structural nature.

These findings align with previous studies [10, 14–16], which reported that tolerant lines often maintain low TYLCDS scores (Fig. 3) despite active viral replication (Fig. 4). Pyramiding of *Ty* genes, particularly *Ty-1*/*Ty-3* and *Ty-2*, was associated with enhanced tolerance and reduced viral titer [12, 31, 43]. While TL1 and TL3 only carried the *Ty-4* gene (Fig. 2), they still showed low TYLCDS scores, suggesting the presence of additional resistance genes or synergistic effects when *Ty-4* is combined with others [24]. For instance, increased tolerance was seen in TL1 (*Ty-1*/*Ty-1* and *Ty-4*); TL4 and TL8 (*Ty-4* and *Ty-6*); and TL11 and TL17 (*Ty-1*/*Ty-3* and *Ty-4*) (Figs. 2 and 3). Interestingly, TL5 showed high TYLCD tolerance despite lacking known *Ty* genes, indicating a potentially novel resistance mechanism. This may involve virus evasion, epistatic effects, or uncharacterized resistance genes. TL5 thus represents a valuable genetic resource, warranting further investigation into alternative resistance pathways beyond the known *Ty* loci.

Tolerant TLs exhibited better vegetative growth with long and un-stunted stems, expanded and uncurled leaves (Fig. 5), less flower drop, and reduced fruit abortion, resulting in higher yield (Fig. 6). Compared to the susceptible ‘Castlerock’, the TLs exhibited significantly higher early yield (by 515.0–1426.2% in 2022 and 80.6–421.5% in 2023), total yield (by1984.3–2916.0% in 2022 and 668.8–1235.5% in 2023), and marketable yield (by 1779.3–2898.9% in 2022 and 557.0–1093.0% in 2023; Fig. 6). Fruits from TL1, TL2, TL3, TL4, TL11, TL12, TL15, and ‘Castlerock’ were suitable for fresh consumption due to their TI > 0.7 and M > 10, following standards from Hernández Suárez et al. [53, 54] and Navez et al. [55].

High values of PCV and GCV (> 20%; TYLCD-S-45, TYLCD-S-90, PL, LA, NPF, EY, TY, MY, Lyc, β-Car, and M), high PCV values and moderate GCV values (NFL and VC), or moderate values of PCV and GCV (10–20%: NPB, AFW, FSI, and TA) (Table 2) indicate high variability, suggesting strong potential for effective phenotypic selection [73]. Moderate PCV values and low GCV values for foliar photosynthesis pigment contents suggest the high environmental impact on these traits [73], corroborated by high ECV values (Table 2). Low PCV and GCV (< 10%) for PD, ED, FF, TSS, and TI (Table 2) indicate limited genetic variability [73]. All traits had *δ*^*2*^_*ph*_ > *δ*^*2*^_*g*_ and PCV > GCV (Table 2), confirming significant environmental influences on trait expression [70]. Nonetheless, for most traits (21 out of 27), *δ*^*2*^_*g*_ formed a larger part of *δ*^*2*^_*ph*_ (Table 2), and PCV/GCV ratios ranged from 1.01 to 1.17, indicating high heritability and genetic stability [70]. These were supported by high *h*^*2*^_*b*_ values ranging from 75.0 to 99.6%. However, Chlor-a, Chlor-b, T-Chlor, T-Car, PD, FF, FT, and VC exhibited wider gaps between PCV and GCV (PCV/GCV = 1.38–1.82, Table 2) and lower *h*^*2*^_*b*_ values (18.41–52.82%; Table 2), indicating lower stability and heritability [73]. Previous studies [9, 10, 12] also reported high *h*^*2*^_*b*_ for TYLCD resistance/tolerance, reinforcing the potential for improvement via phenotypic selection. While traits with high heritability can be improved through direct selection, those with moderate heritability may require more complex breeding due to non-additive gene action [73]. In conclusion, phenotypic selection based on TYLCD severity, vegetative growth (PL, NPB, and LA), plant yield components (AFW, NPF, EY, TY, and MY), and fruit quality traits (ED, FSI, NFL, TSS, TA, Lyc, B-Car, TI, and M) can effectively identify elite TLs for breeding.

Understanding genetic and phenotypic correlations among traits is essential in breeding programs aiming to improve specific traits without adversely affecting others [74]. In this study, most traits exhibited *r*_*g*_ >*r*_*ph*_ (Table 3), indicating moderate underlying genetic correlations [74]. Significant correlations revealed that selecting for low TYLCDS and high TY, AFW, FF, and TSS could reduce VC and NFL, but improve EY, MY, NPF, FED, and β-Carot (Table 3). These trade-offs suggest potential linkage drag, in which resistance genes co-segregate with alleles that adversely affect fruit traits. Zengin et al. [75] reported negative correlations between *Ty-3a*-mediated resistance and both AFW and FPD, likely due to interactions between wild-type resistance genes and fruit-related QTLs on chromosome 2. Similarly, Mahmoud and Osman [10] observed a negative genotypic and phenotypic correlation between TYLCD tolerance and FSI, but a positive genotypic correlation between TYLCD tolerance and both TI and M. Mahmoud et al. [12] also found negative genotypic and phenotypic correlations between TYLCD tolerance and TY, FF, ED, and TSS, along with negative genotypic correlations with VC and TA. The negative correlation between TYLCD-resistance/tolerance and yield is biologically plausible, as TYLCV infection causes stunted growth, flower and fruit drop, and reduced fruit size and weight [1, 14]. These effects may promote the co-inheritance of resistance genes with less desirable traits, particularly in homozygous *Ty* gene carriers, which are sometimes linked to yield penalties. Despite this, the elite TLs in this study exhibited strong TYLCD tolerance with high yields and improved fruit quality, suggesting that linkage drag was effectively minimized through strategic phenotypic selection, likely supported by favorable recombination events. These findings underscore the importance of integrating marker-assisted selection with phenotypic screening to balance resistance and agronomic performance in resistance breeding programs.

Multivariate analyses, including PCA and CA, are commonly used to assess variability and classify germplasms [10, 12, 76]. In this study, PCA reduced the dimension of 27 traits to five PCs, representing 85.07% of the total variance (Fig. 9). These components were significantly influenced by key traits, supporting their effectiveness in distinguishing TLs [77]. This aligns with previous findings [10, 12, 17], which demonstrated PCA’s utility in distinguishing tomato germplasm based on TYLCD severity, vegetative growth, yield, and fruit quality traits. Future evaluations can streamline genotype classification by focusing on a reduced set of informative traits, thereby saving time, labor, and costs with minimal loss of information [77].

The first two PCs accounted for 56.13% of the total variance and captured 19 of 27 estimated traits (Fig. 10), justifying the use of a biplot to examine the relationships among traits and lines [63]. Vectors for TYLCD-S, FSI, Lyc, and β-Car were positioned within adjacent quadrants at angles less than 90° (Fig. 10), indicating positive correlations. Conversely, vectors for the residual traits were arranged in opposing quadrants, also with angles less than 90° (Fig. 10), revealing negative correlations with TYLCD-S. The biplot-based correlations were consistent with the correlation estimates, particularly the phenotypic ones (Table 3).

The biplot revealed that TLs were distributed across all quadrants (Fig. 10), indicating high genetic diversity both within and among groups [77]. Genotypes with more extreme trait values were located farther from the origin, often at the vertices of the convex hull. The most favorable phenotypes for key traits were observed in TL1, TL2, TL4, TL5, TL7, and TL10, whereas ‘Castlerock’ exhibited the poorest performance in TYLCD resistance and plant yield-related components (Fig. 10). TL3, TL8, TL12, TL13, and TL16 clustered near the origin, suggesting a limited contribution to variability under TYLCV-infected conditions (Fig. 10). HCA of morphological traits clustered the TLs into four clusters [62]. Clusters 1 and 3 outperformed Clusters 2 and 4 in TYLCD tolerance, vegetative growth, yield components, and fruit quality, as reflected in population means (Table 5). Lines within Clusters 1 and 3 showed greater potential for selection, and crosses between these clusters may produce F_1_ hybrids with improved tolerance and yield performance.

## Conclusion

The development of TYLCD-tolerant lines supports more sustainable tomato production by reducing reliance on chemical control. Commercial TYLCD-tolerant F_1_ hybrids provide diverse *Ty* resistance genes along with genetic diversity in vegetative growth, yield components, and fruit quality. Segregating hybrid generations were used to develop lines carrying multiple *Ty* resistance genes and improved agronomic performance, offering valuable germplasm for breeding high-yielding, TYLCD-tolerant lines. To ensure their stability and adaptability, further studies should investigate environmental influences on resistance expression and quantify viral loads. These tolerant lines are also promising candidates for marker development, facilitating more efficient selection through marker-assisted breeding. Notably, the absence of known *Ty* genes in some tolerant lines, such as TL5, suggests the presence of novel resistance genes or epistatic interactions, meriting further genetic and molecular investigation. Future research should integrate advanced genomic approaches, including whole-genome sequencing or transcriptomics analyses, to identify novel resistance loci and gene networks underlying TYLCD tolerance.

## Supplementary Information

Supplementary Material 1.

## Data Availability

Data Availability: All data supporting the findings are included within the article, and no additional source data are needed. Materials availability: The advanced tomato lines are available from the corresponding author for non-commercial research purposes, subject to a formal agreement approved and endorsed by the student’s affiliated institution.

## Abbreviations

AES: Agricultural experiment station
AFW: Average fruit weight
ANCOVA: Analysis of covariance
ANOVA: Analysis of variance
β-Carot: β-carotene
chlor-a: Ahlorophyll a
chlor-b: Chlorophyll b
DAT: Days after transplanting
EU: Experimental unit
EY: Early yield
FED: Fruit equatorial diameter
FF: Fruit firmness
FPD: Fruit polar diameter
FSI: Fruit shape index
FT: Flesh thickness
GCV: Genotypic coefficient of variation
h^2^_b_: Heritability
HCA: Hierarchical cluster analysis
LA: Leaf area
Lyc: Lycopene
M: Maturity
MY: Marketable yield
NFL: Number of fruit locules
NPF: Number of plant fruits
NPMB: Number of plant main branches
PCA: Principal component analysis
PCV: Phenotypic coefficient of variation
PL: Plant length
RCBD: Randomized complete block design
r_g_: Genotypic correlation coefficient
r_ph_: Phenotypic correlation coefficient
TA: Titratable acidity
t-carot: Total carotenoids
t-chlor: Total chlorophyll
TEM: Transmission electron microscope
TI: Taste index
TL: Tomato lines
TSS: Total soluble solids
TY: Total yield
TYLCD: Tomato yellow leaf curl disease
TYLCDS: Tomato yellow leaf curl disease severity
TYLCV: Tomato yellow leaf curl virus
VC: Vitamin C
δ^2^_g_: Genotypic variance
δ^2^_ph_: Phenotypic variance
