# Supplementary material for: ExploringTy resistance genes and genetic diversity in improved tomato lines selected from commercial hybrids
Source: BMC Plant Biol. 2025 Sep 23;25:1213. doi: 10.1186/s12870-025-07344-6 (PMC12459071; doi:10.1186/s12870-025-07344-6)
Supplement: Supplementary file 1 — Supplementary Material 1. [file 12870_2025_7344_MOESM1_ESM.docx]

**Table S1. Phenotype of TYLCD-symptomless selected plants of F_5_ families.**

| **The original F_1_ hybrid** | **Code^z^** | **TSS** | **Average fruit weight (g)** | **Early Yield (g plant^-1^)** | **Total yield (g plant^-1^)** | **Flesh thickness (mm)** | **Fruit shape index** | **Fruit firmness (kg/cm^2^)** | **No. of fruit locules** |
| --- | --- | --- | --- | --- | --- | --- | --- | --- | --- |
| **‘Nairouz’** | **F_5:_ 7-R2-8** | 3.5 | 80 | 2800 | 3500 | 0.7 | 0.88 | 3.60 | 3 |
|  | **F_5:_ 7-R2-9** | **3.6** | **90** | **2200** | **4000** | **0.6** | **0.88** | **5.85** | **3** |
|  | **F_5:_ 7-R3-1** | 3.5 | 75 | 3000 | 4000 | 0.6 | 0.88 | 5.85 | 5 |
|  | **F_5:_ 7-R3-2** | 4.0 | 79 | 2600 | 3500 | 0.5 | 0.96 | 4.50 | 4 |
|  | **F_5:_ 7-R3-3** | 4.0 | 80 | 2600 | 3450 | 0.5 | 0.96 | 4.41 | 3 |
|  | **F_5:_ 7-R3-8** | 3.9 | 97 | 1605 | 3505 | 0.5 | 0.93 | 4.05 | 4 |
|  | **F_5:_ 7-R3-9** | **4.0** | **95** | **2000** | **3500** | **0.6** | **0.97** | **5.54** | **4** |
|  | **F_5:_ 7-R3-10** | 4.5 | 76 | 2200 | 3000 | 5.6 | 0.91 | 5.04 | 4 |
| **‘65010’** | **F_5:_ 8-R2-2** | **6.1** | **100** | **500** | **3500** | **0.6** | **0.71** | **5.85** | **5** |
|  | **F_5:_ 8-R2-3** | 3.4 | 115 | 2900 | 4000 | 0.6 | 0.83 | 5.45 | 3 |
|  | **F_5:_ 8-R2-4** | 3.9 | 117 | 1100 | 3800 | 0.5 | 0.71 | 4.14 | 5 |
|  | **F_5:_ 8-R3-3** | 3.5 | 70 | 1500 | 3500 | 0.5 | 0.86 | 6.03 | 4 |
|  | **F_5:_ 9-R2-2** | **4.2** | **97** | **1800** | **3250** | **0.8** | **0.84** | **6.84** | **4** |
|  | **F_5:_ 9-R2-5** | 4.6 | 105 | 900 | 3000 | 0.4 | 0.83 | 4.14 | 4 |
|  | **F_5:_ 10-R1-3** | 3.9 | 86 | 1600 | 3000 | 0.4 | 0.81 | 5.31 | 4 |
|  | **F_5:_ 10-R1-4** | 4.0 | 90 | 1000 | 3200 | 0.4 | 0.88 | 5.31 | 4 |
|  | **F_5:_ 10-R3-1** | 3.1 | 105 | 1900 | 3500 | 0.4 | 0.72 | 6.08 | 5 |
|  | **F_5:_ 11-R2-1** | **5.3** | **100** | **1700** | **3500** | **0.7** | **0.73** | **4.95** | **5** |
|  | **F_5:_ 11-R2-2** | 3.5 | 110 | 2050 | 3000 | 0.6 | 0.72 | 3.60 | 6 |
|  | **F_5:_ 11-R2-3** | **4.0** | **110** | **1000** | **3150** | **0.7** | **0.70** | **4.50** | **4** |
|  | **F_5:_ 11-R2-4** | 3.5 | 105 | 1600 | 3200 | 0.6 | 0.73 | 4.73 | 5 |
|  | **F_5:_ 11-R2-5** | 3.0 | 100 | 1700 | 3300 | 0.6 | 0.70 | 4.64 | 5 |
|  | **F_5:_ 11-R2-6** | 3.6 | 113 | 1500 | 3050 | 0.5 | 0.72 | 4.77 | 4 |
|  | **F_5:_ 11-R2-7** | 3.5 | 110 | 1450 | 3000 | 0.7 | 0.70 | 4.50 | 4 |
|  | **F_5:_ 11-R2-9** | 3.7 | 106 | 2100 | 3500 | 0.6 | 0.73 | 4.82 | 4 |
|  | **F_5:_ 11-R2-10** | 3.5 | 110 | 1800 | 3600 | 0.6 | 0.69 | 4.95 | 5 |
|  | **F_5:_ 11-R2-11** | 3.1 | 115 | 1600 | 3400 | 0.5 | 0.70 | 4.73 | 4 |
|  | **F_5:_ 11-R3-1** | 2.8 | 85 | 600 | 3200 | 0.4 | 0.84 | 4.55 | 6 |
| **‘SV8320’** | **F_5:_ 12-R1-3** | 3.0 | 90 | 2700 | 3400 | 0.5 | 0.70 | 4.95 | 4 |
|  | **F_5:_ 12-R1-4** | 3.6 | 90 | 800 | 3000 | 0.5 | 0.68 | 4.05 | 4 |
|  | **F_5:_ 12-R1-5** | 3.4 | 85 | 1900 | 3000 | 0.4 | 0.78 | 4.50 | 5 |
|  | **F_5:_ 12-R3-1** | **4.0** | **115** | **2300** | **3800** | **0.7** | **0.72** | **5.04** | **5** |
|  | **F_5:_ 12-R3-4** | 3.5 | 105 | 1505 | 3505 | 0.6 | 0.89 | 5.22 | 4 |
|  | **F_5:_ 14-R1-1** | **4.1** | **120** | **1000** | **3500** | **0.7** | **0.73** | **6.89** | **4** |
|  | **F_5:_ 14-R1-2** | **4.0** | **115** | **900** | **3400** | **0.7** | **0.74** | **6.30** | **4** |
|  | **F_5:_ 14-R1-3** | 3.9 | 125 | 1100 | 3500 | 0.7 | 0.72 | 6.08 | 4 |
|  | **F_5:_ 14-R1-4** | **4.0** | **120** | **1300** | **3300** | **0.7** | **0.75** | **5.85** | **4** |
|  | **F_5:_ 14-R1-5** | **4.2** | **120** | **1100** | **3200** | **0.7** | **0.70** | **6.35** | **4** |
|  | **F_5:_ 14-R1-6** | **4.1** | **115** | **1300** | **3400** | **0.7** | **0.71** | **6.39** | **4** |
|  | **F_5:_ 14-R1-7** | 3.9 | 120 | 1500 | 3500 | 0.7 | 0.73 | 6.26 | 4 |
|  | **F_5:_ 14-R1-8** | **4.0** | **125** | **1250** | **3250** | **0.7** | **0.73** | **6.08** | **4** |
|  | **F_5:_ 14-R1-9** | **4.2** | **120** | **1000** | **3350** | **0.7** | **0.73** | **6.53** | **4** |
|  | **F_5:_ 14-R1-10** | **4.5** | **115** | **1600** | **3400** | **0.7** | **0.71** | **6.44** | **4** |
|  | **F_5:_ 14-R1-11** | **4.0** | **115** | **1250** | **3450** | **0.7** | **0.71** | **6.12** | **4** |
|  | **F_5:_ 14-R1-12** | 3.9 | 115 | 1000 | 3000 | 0.7 | 0.71 | 6.71 | 4 |
| **‘Tyrmes’** | **F_5:_ 17-R1-1** | 3.4 | 95 | 1200 | 3000 | 0.5 | 0.83 | 5.40 | 3 |
|  | **F_5:_ 18-R1-1** | **4.0** | **150** | **1500** | **3000** | **0.5** | **0.68** | **5.94** | **5** |

^z^Code: Generation – plant number in replicate.

The selected F_5_ symptomless plants are shaded in green.

**Table S2. Scheme of ANOVA and ANCOVA for pooled data from two years of RCBD.**

| **Source of variance** | **df** | **MS (or MP)** | **Expected MS** | **Expected mean cross products** |
| --- | --- | --- | --- | --- |
| **Year (Y)** | **y-1** | **-** | **-** | **-** |
| **Replicate (R)** | **r-1** | **-** | **-** | **-** |
| **R (Y)** | **y (r-1)** | **-** | **-** | **-** |
| **Genotype (G)** | **l-1** | ***MS_g_*** | ***δ^2^_e_* + r*δ^2^_gy_* + yr*δ^2^_g_*** | ***COV_e_* + r*COV_gy_* + yr*COV_g_*** |
| **G × Y** | **(y-1) (g-1)** | ***MS_gy_*** | ***δ^2^_e_* + r*δ^2^_gy_*** | ***COV_e_* + r*COV_gy_*** |
| **Pooled error** | **y (r-1) (g-1)** | ***MS_e_*** | ***δ^2^_e_*** | ***COV_e_*** |

y: number of years, r: number of replicates, and g: number of germplasm (tomato lines).

*δ^2^_g_*: genotypic variance, *δ^2^_gy_*: interaction variance between germplasm and years; *δ^2^_e_*: error variance; and similarly, for covariances.

| **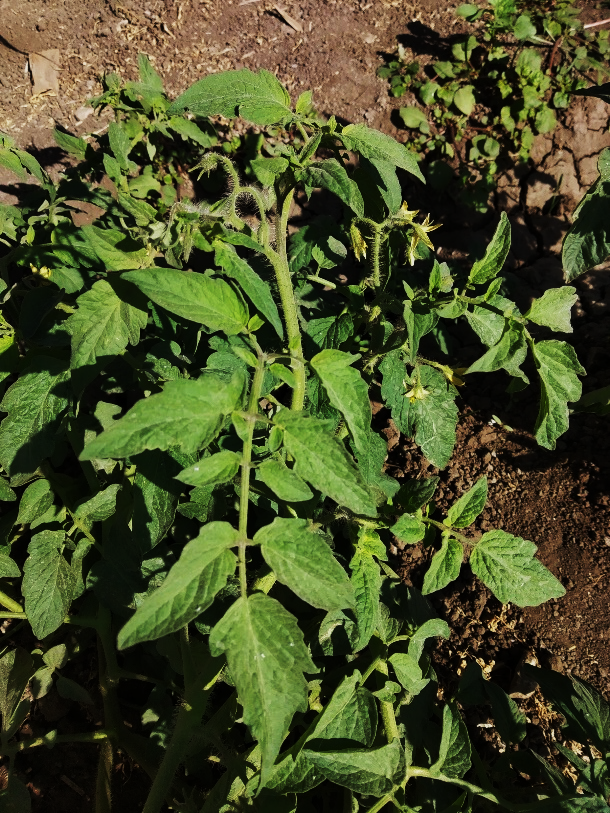**  **Score 1** | **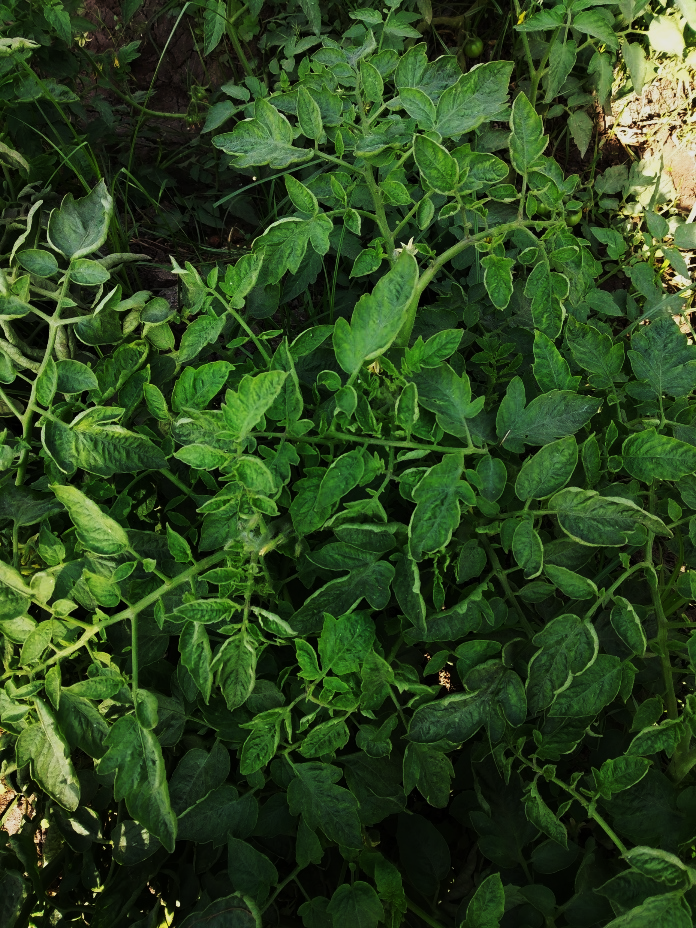**  **Score 2** |
| --- | --- |
| **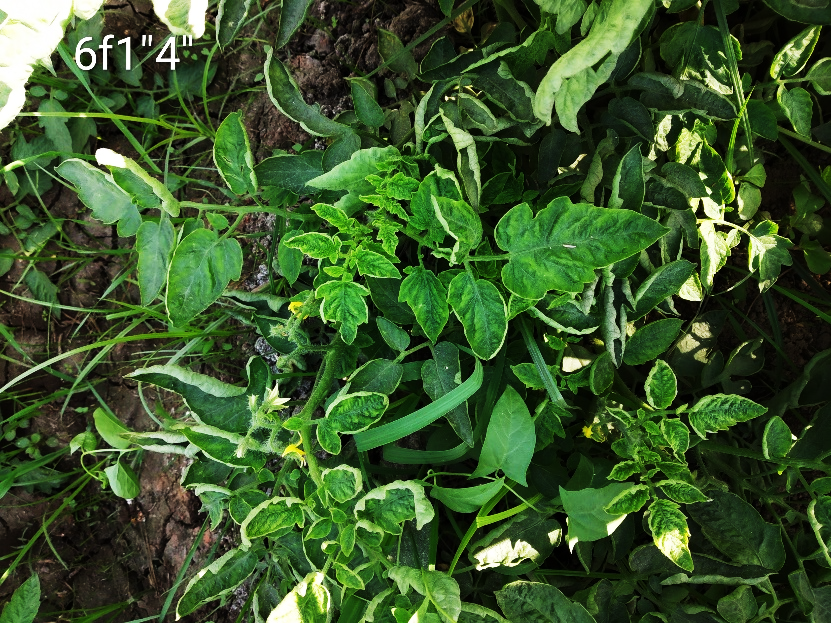**  **Score 3** | **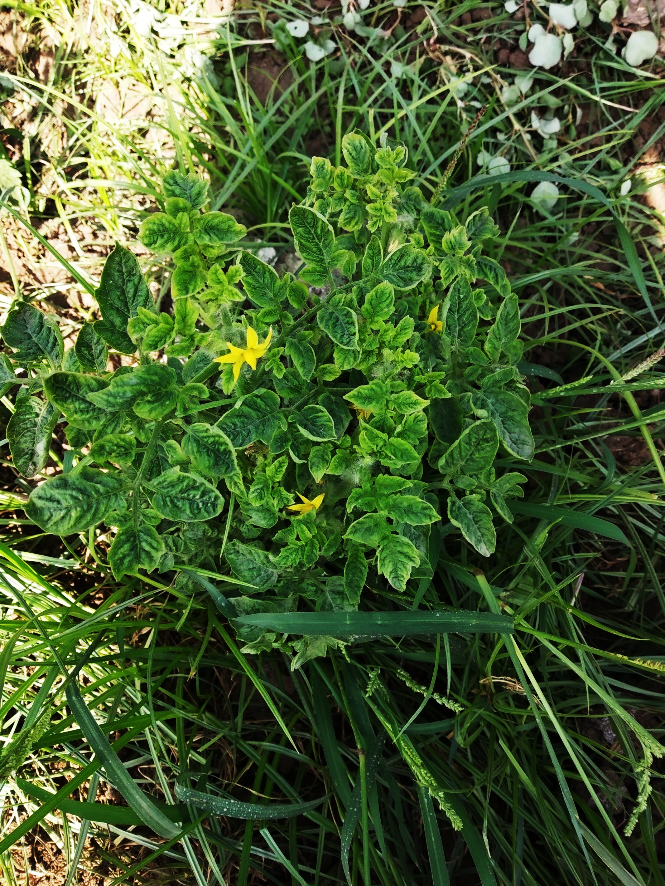**  **Score 4** |
| **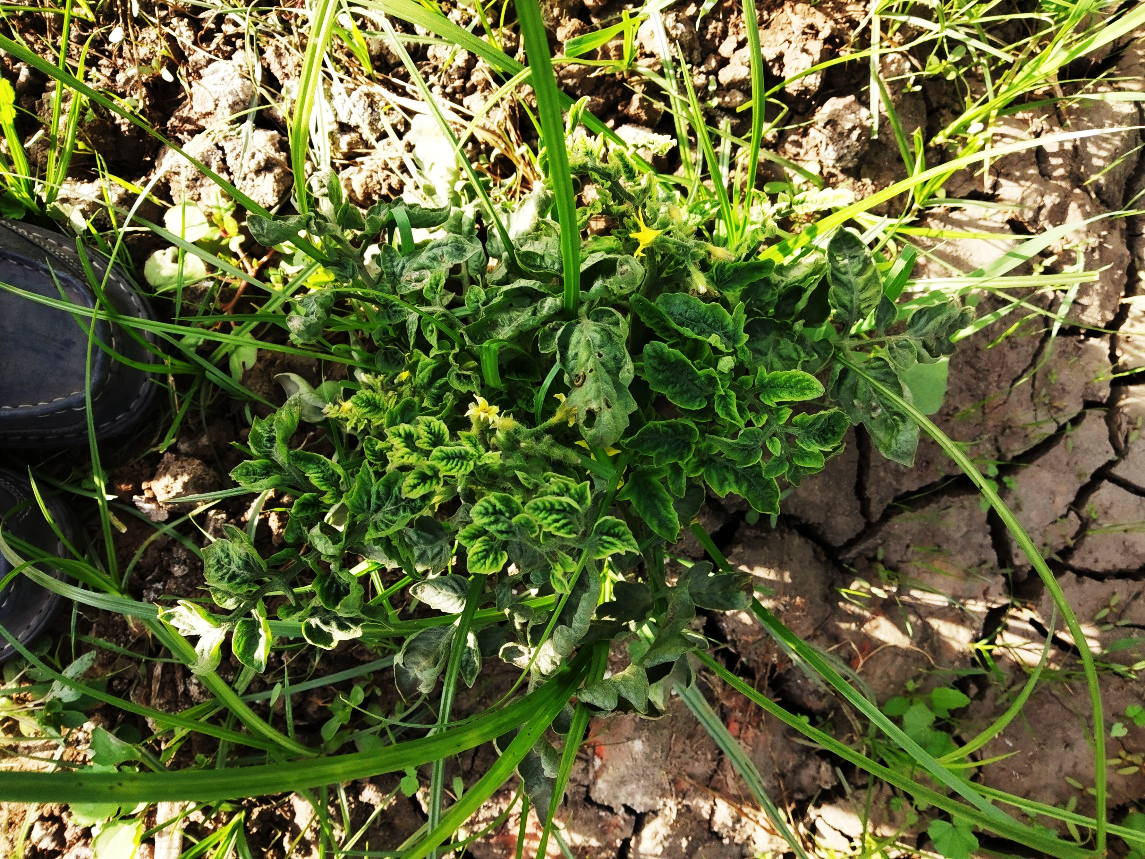**  **Score 5** | |

**Fig. S1. TYLCD symptoms severity score.**

TYLCD severity score was 1: symptomless plant, 2: slight symptoms on the plant top, 3: moderate symptoms, 4: severe symptoms on the entire plant, and 5: severe symptoms and plant stunting.

| 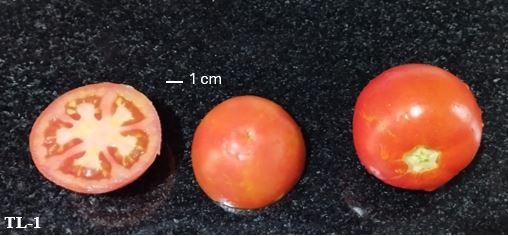 | 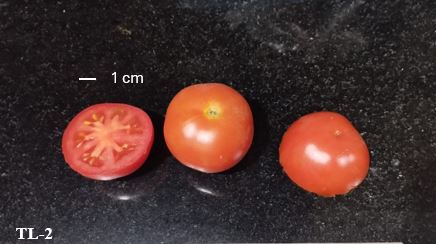 | 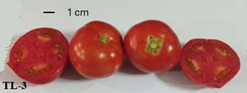 | | 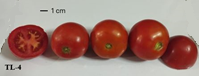 |
| --- | --- | --- | --- | --- |
| 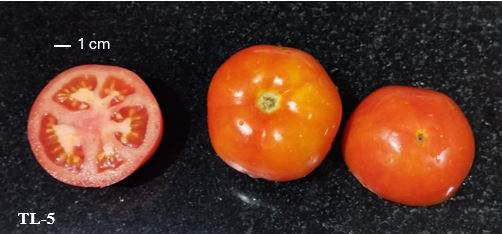 | 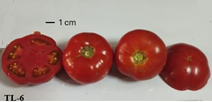 | 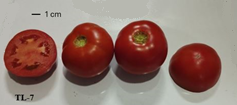 | | 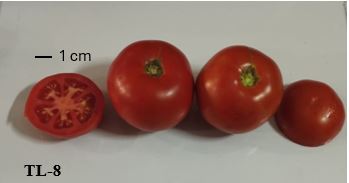 |
| 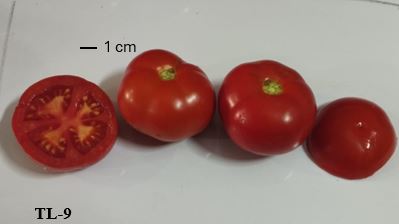 | 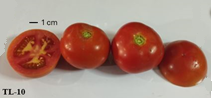 | 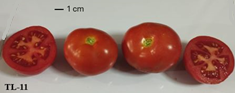 | | 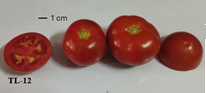 |
| 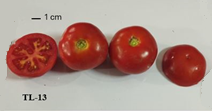 | 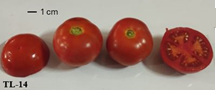 | 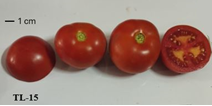 | | 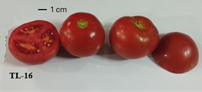 |
| 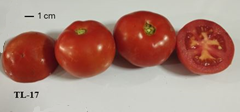 | | | 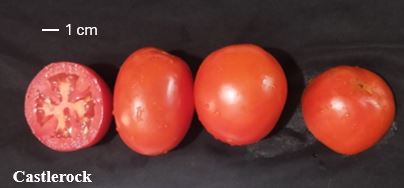 | |

**Fig. S2. Fruits of tomato lines.**

F_7_ tomato lines selected from tomato commercial F_1_ hybrids ‘Nairouz’ for TL-1&TL-2, ‘65010 F_1_’ for TL-3&TL-6, ‘SVTD8320 F_1_’ for TL-7-TL16, and ‘Tyrmes F_1_’ for TL-17.

A photo’s aspect ratios are preserved when zoomed in, highlighting the 1cm mark in each one.
